# Salvage therapies for local recurrence of renal cell carcinoma: a systematic review and meta-analysis

**DOI:** 10.1007/s00345-026-06684-8

**Published:** 2026-08-16

**Authors:** Agata Suleja, Ahmed R. Alfarhan, Marcin Miszczyk, Riccardo Campi, Hussain Albin Hamdhah, Daniele Amparore, Giulio Francolini, Navid Roessler, Keiichiro Miyajima, Shota Inoue, Felix Lübbersmeyer, Abdulrahman A. Alhazeem, Paweł Rajwa, Axel Bex, Alessandro Volpe, Piet Ost, Shahrokh F. Shariat

**Affiliations:** 1 Department of Urology, Comprehensive Cancer Center, Medical University of Vienna, Vienna General Hospital, Währinger Gürtel 18-20, Vienna, 1090 Austria; 2 Department of Urology, Prince Saud Bin Jalawi Hospital, Al Ahsa Health Cluster, Al ahsa, Kingdom of Saudi Arabia; 3 Collegium Medicum, Faculty of Medicine, WSB University, Dąbrowa Górnicza, Poland; 4 Department of Experimental and Clinical Medicine, University of Florence, Florence, Italy; 5 Unit of Urology and Renal Transplantation, Oncology Department, Careggi University Hospital, Florence, Italy; 6 Division of Urology, University of Turin, San Luigi Gonzaga Hospital, Turin, Italy; 7 Radiation Oncology Unit, Oncology Department, Azienda Ospedaliero Universitaria Careggi, Florence, Italy; 8 Department of Urology, Medical University Center Hamburg-Eppendorf, Hamburg, Germany; 9 Department of Urology, The Jikei University School of Medicine, Tokyo, Japan; 10 Department of Urology, Dentistry and Pharmaceutical Sciences, Okayama University Graduate School of Medicine, Okayama, Japan; 11 Department of Urology, King Fahad Hospital Hofuf, Al Ahsa Health Cluster, Al Ahsa, Kingdom of Saudi Arabia; 12 Division of Surgery and Interventional Sciences, University College London and University College London Hospital, London, UK; 13 Second Department of Urology, Centre of Postgraduate Medical Education, Warsaw, Poland; 14 Department of Urology, Royal Free Hospital, London, UK; 15 The Netherlands Cancer Institute, Amsterdam, The Netherlands; 16 Università degli Studi del Piemonte Orientale “Amedeo Avogadro”, Vercelli, Italy; 17 Cancer Research Institute Ghent (CRIG), Ghent University, Ghent, Belgium; 18 Department of Human Structure and Repair, Ghent University, Ghent, Belgium; 19 Department of Radiation Oncology, Iridiumnetwerk, Antwerp, Belgium; 20 Department of Urology, University of Texas Southwestern Medical Center, Dallas, TX USA; 21 Hourani Center for Applied Scientific Research, Al-Ahliyya Amman University, Amman, Jordan; 22 Department of Urology, Weill Cornell Medical College, New York, NY USA; 23 Department of Urology, Second Faculty of Medicine, Charles University, Prague, Czech Republic; 24 Karl Landsteiner Institute of Urology and Andrology, Vienna, Austria

**Keywords:** Renal cell carcinoma (RCC), Partial nephrectomy (PN), Radical nephrectomy, Salvage treatment, Local recurrence, Ablative therapies, Radiofrequency ablation, Cryoablation, Stereotactic body radiotherapy (SBRT), Nephron-sparing surgery

## Abstract

**Background:**

We aimed to systematically summarize data on efficacy of local salvage therapies for local recurrence of renal cell carcinoma (RCC).

**Methods:**

We searched MEDLINE, Embase, Web of Science, CENTRAL, and Google Scholar through 05/08/2026, for studies on local salvage therapies for local recurrence of RCC following radical nephrectomy, partial nephrectomy, or ablation (PROSPERO: CRD42024623099). Meta-analyses were conducted using random-effects models. Risk of bias (RoB) was assessed using ROBINS-I.

**Results:**

Forty-seven studies comprising 2039 patients were included (45 retrospective). Patients recurring after primary ablation predominantly presented with new renal masses, and were managed with repeat ablation (6 studies, *n* = 193) or salvage surgery (3 studies, *n* = 48), both yielding low recurrence rates. Local recurrences (M0) after primary partial nephrectomy (PN), most often in patients with pT1 disease, were treated with repeat PN, radical nephrectomy (RN), or ablation (15 studies, *n* = 562), with heterogeneous outcomes across modalities, ranging from low recurrence rates to predominantly distant metastatic failure. In contrast, recurrences after primary RN (12 studies, *n* = 564) occurred in a population with primarily locally advanced disease (pT3-4) and outcomes were notably worse, driven by distant progression. Four studies used radiotherapy in salvage setting, with heterogeneous methods and outcomes. Majority of studies were assessed moderate RoB, 16 studies assessed as serious, predominantly due to bias in participant selection.

**Conclusion:**

Efficacy of local salvage therapies for RCC recurrence is influenced by the primary treatment and histopathological advancement of primary disease. Repeat ablation provides favorable outcomes, while recurrences after PN can be managed with repeat surgery or ablation, with heterogeneous results (from low recurrence rates to frequent distant metastases). Post-RN patients, often with initially advanced disease, have worse prognosis with approximately two-thirds experiencing progression by three years, primarily due to distant metastases. Overall, quality of evidence remains low. There is critical need for expert consensus on quality indicators for locally recurrent RCC care and for prospective registries with standardized outcome reporting.

**Supplementary Information:**

The online version contains supplementary material available at 10.1007/s00345-026-06684-8.

## Background

Treatment of localized and locally advanced renal cell carcinoma (RCC) comprises several techniques [1]. Partial nephrectomy emerged as the preferred approach for stage T1a tumors (≤ 4 cm) when technically feasible, with its use extending to T1b (4–7 cm), or even selected stage II-III disease patients [2]. For T1a-T1b disease, alternative treatment options include ablative techniques (e.g., radiofrequency ablation [RFA], cryoablation, microwave ablation, or stereotactic body radiotherapy [SBRT]), offering renal function preservation and comparable oncological outcomes to surgery in carefully selected patients [3]. Active surveillance is also a consideration for small renal masses, particularly in elderly or comorbid patients. Radical nephrectomy remains a mainstay treatment option for locally advanced disease, especially when the cancer involves vascular structures or adjacent organs [4].

Local recurrence occurs in two primary scenarios: in the renal fossa following radical nephrectomy, or within the remaining renal parenchyma after partial nephrectomy or minimally invasive procedures [5]. This definition can also be extended to include regional recurrence, encompassing ipsilateral regional structures such as retroperitoneal lymph nodes, adrenal gland, and psoas muscle [6]. Retrospective series suggest that five-year recurrence rates in patients treated with surgery are as low as 4.2 to 13.1% [7, 8]; however, ablative therapies can be associated with up to four times higher local recurrence rates compared to partial nephrectomy [9]. For patients treated with SBRT, early outcomes are excellent, but longer follow-up data are missing [10].

A previous systematic review indicated that the majority of available data on salvage therapies comprises moderately small, non-comparative case series, and no randomized controlled trials exist to compare the safety and efficacy of available modern options. However, failure to effectively treat a local recurrence can result in progression to systemic disease, with subsequently poor prognosis and necessity for systemic therapy [11]. As new studies are emerging, we sought to summarize the data on effectiveness and safety of local therapies for local recurrence of RCC, stratified by primary treatment modality.

## Methodology

### Protocol and search strategy

This systematic review and meta-analysis was prospectively registered on PROSPERO (CRD42024623099), and is reported in accordance with PRISMA 2020 and AMSTAR-2 checklists (Supplementary Tables 1–2, Supplementary Appendix 1) [12, 13]. We searched MEDLINE (via PubMed), Embase (via Embase.com), and Web of Science Core Collection databases, Cochrane Central Register of Controlled Trials (CENTRAL; via Ovid), and Google scholar search engine (top 200 hits retrieved) on November 20, 2024, updated on January 20, 2026, and updated a second time on August 5, 2026. No language or publication date filters were imposed. The search strategy was developed using previously described methodology [14], and aimed to identify studies evaluating salvage treatments for definitive treatment of locally recurrent RCC (Supplementary Appendix 2). Following deduplication, abstract screening was performed independently by two investigators (A.S. and A.A.). Included records were retrieved and subjected to full-text screening by the same two independent reviewers (A.S. and A.A.), and reasons for exclusion were noted. Deduplication and both stages of record screening were performed using the Rayyan.ai platform [15]. Selected publications underwent backward citation searching. Any disagreements were resolved through discussion among the authors and a third reviewer (M.M.).

### Study selection

The research question was defined using the PICOS (Population, Intervention, Comparator, Outcome, Study type) framework (Supplementary Table 3). We included studies on patients with isolated local recurrence of RCC in previously treated kidney, renal fossa, regional lymph nodes, adrenal gland, or psoas muscles who were treated with local definitive salvage therapy. This composite definition of isolated loco-regional recurrence, which refers to renal fossa and remaining renal parenchyma together with the ipsilateral adrenal gland, retroperitoneal lymph nodes, and psoas muscle, follows the convention applied in contemporary RCC guidelines and in previously published institutional series of locally recurrent RCC [1, 6]. Patients with synchronous distant metastases (M1) were excluded throughout. Studies primarily assessing local treatments of distant metastases or treatments in contralateral kidney were excluded. Studies comprising mixed populations were included if it was possible to separately extract data for patients relevant to this review. Both comparative and non-comparative studies were considered eligible. The main outcome of interest was recurrence-free survival (RFS). Secondary outcomes included overall survival (OS), cancer specific survival (CSS), and rates of serious (grade ≥ 3) adverse events (AEs). RFS was defined as time from salvage treatment until local or systemic recurrence. Studies reporting on similarly defined endpoints, regardless of the naming convention (e.g. progression-free survival or disease-free survival), were included. Endpoints accounting for only one type of recurrence (local or distant) were excluded from the meta-analysis. Both retrospective and prospective studies were included. Review articles, letters to editors, editorial comments, and case series comprising less than 10 relevant patients were excluded. Conference abstracts were considered eligible.

### Data extraction and risk of bias analysis

Extracted data included: type of study, sample size, clinical characteristics of the patients, median follow-up, details of the salvage treatment, rates and probabilities for the outcomes of interest. Data were extracted independently by two authors (A.S. and A.A., with H.H.), and conflicts were mediated by a third author (M.M.). Risk of bias (RoB) was evaluated independently by two authors for each study using the ROBINS-I tool [16].

### Statistical analysis

Meta-analyses were conducted using the “metaprop” function in “meta” package in R (version 4.4.2; developed by the R Foundation for Statistical Computing in Vienna, Austria), in accordance with Cochrane Collaboration guidelines [17]. For the purpose of meta-analysis, rates of severe or worse treatment-related AEs, and time-dependent outcomes at given timepoints (3-year RFS and 5-year OS/CSS), were assessed as proportions. For AE rates, the numerator represented the number of patients who experienced specified AE. For time-dependent outcomes, the numerator was calculated by multiplying the total number of patients and Kaplan-Meier estimated survival probability at given timepoint of interest, and rounding the result to the nearest integer. The denominator corresponded to the total number of patients assessed for each outcome. When such information was not directly reported in the source, we sought to retrieve necessary data from available graphs using the WebPlotDigitizer software [18]. Pooled estimates were calculated using random-effects generalized linear mixed models (GLMM), with random intercept logistic regression approach, employing a logit transformation for proportions and maximum-likelihood estimation for between-study variance (τ²). Heterogeneity was evaluated using Cochran’s Q and *I*^2^ test. Individual study 95% confidence intervals (CI) were calculated using the Clopper-Pearson method, with a continuity correction of 0.5 applied to studies with zero events solely for individual CI calculation. Pooled RFS, OS, CSS, and AE rates with corresponding 95% confidence intervals (CI) were visualized on forest plots, with subgroups according to the primary treatment method and salvage modality - no pooling was performed across dataset as whole; no statistical comparison was made between subgroups. Sensitivity analyses were performed to account for heterogeneity and RoB. To explore whether 5-year OS in the post-radical nephrectomy salvage surgery subgroup varied with publication year, an exploratory random-effects meta-regression using the metareg function, with publication year (centered on the median) as a single moderator was performed. Publication bias was assessed in analyses including ≥ 10 inputs per subgroup via funnel plots and Egger’s test. P-values at < 0.05 were considered significant, and all tests were two-sided.

## Results

Following the screening of 5866 individual records, we selected 45 retrospective studies (*n* = 1932) published between 2003 and 2026, one prospective registry study (*n* = 81) [19], and one single-arm phase II trial (*n* = 26) [20] (Supplementary Fig. 1). The RoB analysis is summarized in Supplementary Table 4.

### Salvage surgery after radical nephrectomy

Twelve studies comprising 564 patients described outcomes of salvage surgery after radical nephrectomy [21–32] (Table 1). The study cohorts often presented with multiple advanced disease features at primary treatment, including locally advanced (pT3-4, in 27–69% of patients) or regional (N1) disease and large median lesion sizes (5 to 9 cm), together with regional recurrence at salvage treatment (Table 1). None of the studies included patients with systemic disease or cytoreductive nephrectomies. The majority of included studies were at moderate RoB, primarily due to retrospective study design and bias in participant selection criteria [21–24, 26, 27, 31, 32]. Four studies were assessed as having serious RoB, due to bias in selection of participants, as they allowed inclusion of patients who had received systemic therapy before surgery, which could interfere with assessment of local treatment’s efficacy [25, 28–30].

**Table 1:** Summary of studies on surgical methods for local salvage treatment of recurrent renal cell carcinoma following radical nephrectomy

Author Year	Time	*N* patients (%)	Median age [years]	Male (%)	Median time to recurrence [months]	Median follow-up [months]	pTNM-T Staging (%)					TNM-*N* Staging	ccRCC (%)	Median Tumour Size [cm]		Prior systemic therapy (%)	Location (%)	Oncological Outcomes
							pT1	pT2	pT3	pT4	pTX			Primary	At Salvage			
Primary treatment with radical nephrectomy (*n* = 564)																		
Candia et al. 2024 [80]	2016–2023	13	74	10 (77%)	67	30	11 (85%)	1 (8%)	1 (8%)	0	0	NA	13 (100%)	5.3	2.2	15	LR (23%) RR (77%)	3-year RFS was 85%, with two patients experiencing distant recurrence. All patients were alive at the end of FU. No severe AEs were recorded.
Huang et al. 2023 [23]	2008–2020	182	NA	137 (75%)	25	35	104 (57%)		78 (43%)		0	N0X = 170 N1 = 12	149 (82%)	5	3.3	NA	LR (65%) RR (35%)	3-year RFS and CSS were 42% and 76%, respectively. 5-year RFS and CSS were 27% and 69%, respectively. Majority (89%) of recurrences involved distant metastases.
Staník et al. 2022 [24]	2004–2019	55	64	37 (67%)	42	50	18 (33%)	9 (16%)	19 (35%)	0	9 (16%)	N0 = 1 N1 = 6 NX = 48	39 (71%)	NA	4.2	NA	LR (31%) RR (69%)	Median RFS was 25 months, with local recurrence in 20%, and systemic progression in 42% of the patients. Five-year OS was 62%.
^^^Liu et al. 2022 [25]	2001–2020	34	43	23 (68%)	14.6	25.8	20 (59%)		14 (41%)		0	N0 = 27 N1 = 7	17 (50%)	NA	3.2	43.8**	LR (29%) RR (71%)	3-year PFS and OS were 22% and 78%, respectively. 5-year OS was 55%.
Romeo et al. 2020 [26]	2010–2016	13*	61	12 (92%)	29.7	18	3 (23%)	1 (8%)	7 (54%)	2 (15%)	0	N0 = 11 N1 = 2	10 (77%)	6.5	NA	NA	LR and RR	3- and 5-year CSS were 92% and 70%, respectively. Metastatic progression occurred in 54% of the patients.
Herout et al. 2018 [81]	1992–2014	54	65	39 (72%)	36	48	21 (39%)	11 (20%)	20 (37%)	2 (4%)	0	N0/X = 50 N1 = 4	43 (80%)	6.8	4.2	NA	LR (87%) RR (13%)	3- and 5-year OS were 72% and 60%, respectively. Half of the patients developed metastases. One patient experienced G3 AE.
Thomas et al. 2015 [28]	1990–2014	102	55	29 (28%)	19	32	20 (20%)	20 (20%)	59 (58%)	3 (3%)	0	N0/X = 82 N1 = 20	66 (65%)	8	4.5	45.1	LR (48%) RR (52%)	3-year RFS and CSS were 40% and 71%, respectively. 5-year RFS and CSS were 32% and 52%, respectively. Majority (83%) of recurrences involved distant metastases. Two patients died of postoperative multi-organ failure.
Fayek et al. 2014 [29]	2004–2012	22	60.1^#^	16 (73%)	13.5	7.5	8 (36%)	5 (23%)	7 (32%)	2 (9%)	0	N0 = 17 N1 = 5	17 (77%)	> 10 (9 Pt) < 10 (13 Pt)	8	NA	LR (59%) RR (41%)	Salvage treatment was complete only in nine patients (41%). Median OS and DFS were 15 and 10 months, respectively.
Margulis et al. 2009 [82]	1990–2007	54	54.5	44 (81%)	NA	41	10 (19%)	11 (20%)	33 (61%)	0	0	N0/X = 43 N1 = 11	NA	9	6	50	LR (65%) RR (35%)	3-year RFS and OS were 28.7% and 62.7%, respectively. 5-year OS was 51.4%. Two patients died of multi-organ failure during perioperative period, and eight experienced grade III/IV AEs.
Bruno et al. 2006 [31]	1989–2004	11	NA	NA	18.1	38.8	5 (45%)	2 (18%)	3 (27%)	0	1(9%)	NA	NA	NA	NA	NA	LR (45%) RR (55%)	At the end of follow-up, three patients had no evidence of disease, three were alive with metastases, and five died of disease.
Sandhu et al. 2005 [32]	1994–2004	14	57.9	12 (86%)	26.6	12	1 (7%)	7 (50%)	5 (36%)	0	1 (7%)	N0/X = 10 N1 = 4	NA	NA	NA	NA	NA	3- and 5-year DFS were 36% and 18%, respectively. 5-year OS was 48%. All patients with recurrence developed metastases.
Göğüş et al. 2003 [21]	1994–2002	10	51.7	7 (70%)	33.6^#^	16.6	1 (10%)	5 (50%)	4 (40%)	0	0	N0 = 10	9 (90%)	NA	8.5	NA	LR (100%)	One patient died due to postoperative complications, two patients died due to metastases (at 3 and 14 mo). Remaining patients were disease-free and alive at the end of FU.

*AEs *Adverse events, *ccRCC *clear cell renal cell carcinoma, *CSS *Cancer Specific Survival, *DFS *Disease Free Survival, *LR *local recurrence (renal fossa or remaining renal parenchyma), *N *number, *NA *Not Available

*OS *Overall Survival, *PFS *Progression Free Survival, *LRFS *local recurrence-free survival, *RFS *recurrence-free survival, *RR *regional recurrence (retroperitoneal lymph nodes, adrenal gland, or psoas muscles)

^#^Mean *One patient treated with cryoablation **Calculated for whole cohort ^clinical stage reported for primary treatment

**Table 2:** Summary of studies on surgical methods for local salvage treatment of recurrent renal cell carcinoma following partial nephrectomy, ablative local treatments, or unselected treatments

Author Year	Time	*N* patients (%)*	Median age [years]	Male (%)	Median time to recurrence [months]	Median follow-up [months]	pTNM-T Staging (%)					TNM-*N* Staging	ccRCC (%)	Median Tumor Size [cm]			Prior systemic therapy (%)	Location (%)	Oncological Outcomes
							pT1	pT2	pT3	pT4	pTX			Primary		At Salvage			
A. Salvage surgery after primary treatment with partial nephrectomy (*n* = 410)																			
Zhou et al. 2026 [40]	2014–2025	sRN = 37 sPN = 14 IT = 4	62.5^p^/60.0^no^	35 (64%)	25.0^o^/31.0^no^	NA	55 (100%)	0	0	0	0	NA	NA	3.5^o^/2.6^no^	NA		NA	LR	Secondary recurrence 8 (14.5%); distant metastasis 4 (7.3%); CSD 6 (10.9%); no difference in RFS/OS between original-site and non-original-site recurrence (*p* = 0.31; *p* = 0.16); pT upstaging at salvage 84.3% vs. 21.7% (*p* < 0.001)
^^^Ma et al. 2024 [33]	2013–2023	sPN = 4 sRN = 11	58	14 (93%)	36	24	14 (93%)	1 (7%)	0	0	0	NA	14 (93%)	NA	3		NA	LR	No local failures or cancer-related mortality were recorded. One patient (7%) had recurrence with distant metastases.
Okhawere et al. 2023 [34]	2012–2020	sPN = 36 sRN = 22	66	39 (67%)	NA	7	34 (59%)	0	9 (16%)	2 (3%)	13 (22%)	NA	14	NA	2.5		NA	LR	Recurrence occurred in 5% of patients treated with sRN, and 3% with sPN; however, follow-up was very short. Major complications occurred in 5% and 6%, respectively.
^^^Di Maida et al. 2023 [35]	2017–2023	sPN = 26	63	18 (69%)	38	37	20 (77%)	3 (12%)	3 (12%)	0	0	NA	NA	NA	3.5		NA	LR	Recurrence occurred in five patients (19%), including three cases of local failure, and two cases of systemic progression. Two patients died over the course of follow-up, including one disease-related death (4%).
Huang et al. 2023 [83]	2008–2021	sPN = 34	57.5	29 (85%)	36	27	33 (97%)	0	1 (3%)	0	0	NA	32 (95%)	3	2.6		NA	LR	3- and 5-year RFS were 78% and 76%, respectively. No significant differences in disease control were found between sPN and sRN.
		sRN = 106	60	81 (76%)	25		98 (92%)	4 (4%)	4 (4%)	0	0		93 (88%)	3.6	3		NA		
^A^Brassetti et al. 2023 [37]	2012-NA	sPN = 15	67	11 (73%)	38	21	NA					NA	NA	4.5	2		NA	LR	Local recurrence occurred in one patient (7%). There were no cases of systemic progression or disease-related mortality
Brassier et al. 2022 [19]	2013–2019	sPN = 8 sRN = 31	66	31 (79%)	21	21	Summary data for surgery and ablation: pT1 (70%), pT2 (6%), pT3 (24%)					NA	34 (87%)	4	3.1		NA	NA	Local recurrence occurred in eight patients (21%), and systemic progression in 18 (46%). There were two G3 and seven G4 complications.
^A^Antonelli et al. 2016 [84]	1983–2015	sRN = 15	NA	NA	42	42	NA					NA	NA	NA	NA		NA	LR	Seven patients died (47%) and four are alive with metastases (27%) at the end of follow-up.
Johnson et al. 2008 [85]	1992–2006	sPN = 47	44	33 (65%)	50	56	NA					NA	NA	NA	3.5		NA	LR	Majority (94%) of patients had VHL. Local recurrence occurred in 20% of operated renal unit. Three patients developed metastases (6%). Ten patients (20%) had major perioperative complications, including one case of perioperative mortality.
B. Salvage surgery after primary treatment with ablation (*n* = 48)																			
^A^Hemal et al. 2024 [41]	2017–2023	sPN = 10	NA	NA	24	16	NA					NA	10 (100%)	2.7	2.3		NA	LR	One patient experienced local recurrence during follow-up. No serious complications were reported.
Margue et al. 2022 [86]	2013–2020	sPN = 11	54	5 (45%)	16	12	NA					NA	NA	NA	3.4		NA	LR	Local recurrence occurred in one patients (9%), and systemic progression in two (18%), all of whom suffered from VHL. Major postoperative complications occurred in 18%.
Jiménez et al. 2016 [43]	1997–2013	sPN = 14 sRN = 13	64	18 (67%)	13	14.5	NA					N1 = 1	19 (70%)	NA	3.6		NA	LR	There was one local (4%) and one systemic (4%) recurrence. Seven patients were lost to follow-up (26%). Six patients reported G3-4 complications.
C. Salvage surgery after heterogeneous initial treatments (*n* = 543)																			
Porreca et al. 2025 [87]	2015–2024	66 (PN + RN)	64^W^	73.3^W^	18^W^	62^W^	NA	NA	NA	NA	NA	NA	NA	4.2^W^	NA		NA	LR	3-year LRFS was 86.4%, the 3- and 5-year CSS was 94,8% and 92.7%, 3- and 5-year OS was 97.5% and 85.5%,
Gaas et al. 2025 [45]	2007–2024	PN = 23 RN = 20 AB = 5	59.4	29 (60%)	23.5	68.2	31 (65%)	7 (15%)	10 (21%)	0	0	NA	44 (92%)	> 4 (31 Pt) < 4 (17 Pt)	> 4 (19 Pt), < 4 (29 Pt)		NA	LR/RR	5-year PFS and CSS were 83% and 86%, respectively. Five patients had G3-4 AEs, and one patient died due to perioperative complications.
^A^Saini et al. 2023 [88]	2013–2022	PN = 6 AB = 12	65	NA	24	15	NA					NA	NA	NA	NA		NA	LR	No recurrences were observed during follow-up. None of the patients developed major postoperative complications.
Beksac et al. 2021 [47]	2010–2020	PN = 58 AB = 14	63.2	56 (78%)	23.2	28.5	41 (57%)	2 (3%)	9 (13%)	0	0	NA	39 (54%)	NA	3.7		NA	LR	Eleven patients (15%) experienced distant progression, including one (1%) with local progression. Major complications occurred in 8%.
Kim et al. 2021 [54]	2007–2013	PN = 58 RN = 97	NA	72 (46%)	NA	32.7	NA					NA	153 (99%)	NA	NA		NA	LR/RR	3-year OS and CSS were 97%. The 5-year OS and CSS were 93% and 94%, respectively.
Barboza et al. 2020 [48]	2011–2017	PN = 2 RN = 17	60	13 (68%)	10.4	31.53	6 (32%)		10 (53%)	1 (5%)	2 (11%)	N0/X = 15 N1 = 4	10 (53%)	7.1	2.6		NA	RR (100%)	Only patients treated with salvage lymphadenectomy were included. 3-year RFS and CSS were 30% and 82%, respectively. 5-year RFS and CSS were 30% and 61%, respectively. Majority of relapses were distant metastases (73%). There was one G3 and one G5 AEs.
Ghandour et al. 2020 [49]	2015–2018	PN = 3 RN = 9	65.5	10 (83%)	48.5	19	NA					NA	11 (92%)	NA	2.97		NA	LR (25%) RR (75%)	Five patients had systemic recurrence (42%), and no patients recurred locally.
^A^Capogrosso et al. 2017 [89]	NA	PN = 16 RN = 21	NA	NA	18	NA	14 (38%)	8 (22%)	14 (38%)	1 (3%)	0	Negative	NA	NA	NA		NA	NA	3- and 5-year DFS were 85% and 67%, respectively. 5-year CSS was 75%.
Du et al. 2016 [51]	1990–2014	PN = 47 RN = 44	63	55 (60%)	29.8	73	51 (56%)		35 (38%)		0	N0 = 84 N1 = 3 M1 = 5	62 (68%)	4.8	NA		NA	LR (88%) RR (12%)	5-year PFS was 32%. Events comprised 41% distant metastases, 20% local recurrence, and missing data for remaining patients. 5-year CSS was 71%. Grade III postoperative complications occurred in four patients, and grade IV in 18 patients.
Abarzua-Cabezas et al. 2015 [90]	2004–2012	PN = 5 AB = 8	64	11 (85%)	NA	32.9^#^	10 (77%)	0	3 (23%)	0	0	NA	8 (62%)	4	NA		NA	NA	One patient experienced recurrence (metastases) during follow-up.
^A^Riedl et al. 2011 [91]	1982–2010	1 2	NA	NA	45.6^#^	36^#^	5 (42%)	0	6 (50%)	0	0	N0 = 11 N1 = 1	NA	NA	NA		NA	NA	Surgery combined with intraoperative and adjuvant radiotherapy. By the end of follow-up, six patients died of disease progression and three were alive with metastases.

*AEs *Adverse events, *ccRCC *clear cell renal cell carcinoma, *CSS *Cancer Specific Survival, *DFS *Disease Free Survival, *LR *local recurrence (renal fossa or remaining renal parenchyma), *N *number, *NA *Not Available

*OS *Overall Survival, *PFS *Progression Free Survival, Recurrence Free Survival recurrence-free survival, *RR *regional recurrence (retroperitoneal lymph nodes, adrenal gland, or psoas muscles); *o *original-site recurrence; *no *non-original-site recurrence

^#^mean; *subset values refer to salvage treatment for primary treatment with partial nephrectomy or ablation, or to primary treatment for studies reporting on mixed treatments ^clinical stage reported for primary treatment ^A^report published as a conference abstract ^W^data for the whole cohort

**Table 3:** Summary of studies on ablative local salvage treatments of locally-recurrent renal cell carcinoma

Author Year	Time	*N* patients (%)*	Median age [years]	Male gender (%)	Median time to recurrence [months]	Median follow-up [months])	pTNM-T Staging (%)	TNM-*N* Staging	ccRCC (%)	Median Tumour Size [cm]		Prior systemic therapy (%)	Location (%)	Oncological Outcomes
										Primary	At Salvage			
A. Salvage ablation after primary treatment with partial nephrectomy (*n* = 152)														
Vaccaro et al. 2025 [55]	2008–2022	27	65	22 (81%)	50	32	NA	NA	20 (74%)	NA	2.2	NA	LR	3- and 5-year RFS was 64%. Majority of recurrences were distant (67%). One patient had G3 perioperative complication.
Krajewski et al. 2024 [59]	NA	23	67.1^#^	7 (30.4)	23	NA	NA	NA	8 (52.2%**)	2.1	NA	NA	LR (52.2) RR (26.1) RES (21.7)	Four patients with nonradical ablation, three patients with LR contrast uptake at the ablation site. During follow-up two non-cancer related deaths, no metastatic spread
Brassier et al. 2022 [19]	2013–2019	42	69	31 (74%)	20	22	Summary data for surgery and ablation: pT1 (70%), pT2 (6%), pT3 (24%)	NA	34 (81%)	4	2.1	NA	LR	Eight patients developed a local recurrence (19%), and four systemic (10%).
^A^Abdelsalam et al. 2020 [56]	2005–2019	35	66	NA	NA	48	NA	NA	21 (60%)	2.3	NA	NA	LR	Three lesions recurred locally (6%). Four patients had G3 complications.
Zhou et al. 2018 [57]	2006–2017	PN = 8 RN = 3	76	8 (73%)	48	30	NA	NA	8 (73%)	NA	2.8	NA	LR	One patient had local recurrence (9%) and two patients died to unrelated causes (18%). No serious AEs were recorded.
Yang et al. 2013 [58]	2003–2010	14	45	8 (57%)	NA	37.6	NA	NA	5 (36%)	NA	2.64^#^	NA	LR	All patients had VHL. Four patients had local recurrence (29%), and none systemic. No serious AEs were recorded.
B. Salvage ablation after primary treatment with ablation (*n* = 193)														
^A^Das et al. 2023 [92]	NA	13	68	NA	NA	19	NA	NA	NA	3.7	NA	NA	LR	One patient had recurrence of disease (8%).
Loloi et al. 2020 [61]	2010–2015	50	NA	NA	13.7^#a^	28^a^	NA	NA	66%^a^	3.1^a^	NA	NA	LR	Twelve patients (24%) had local recurrence, nine of whom proceeded to next salvage treatment.
Sundelin et al. 2019 [62]	2005–2017	72	66.5	46 (64%)	6 (*n* = 42) 19 (*n* = 30)	23	NA	NA	55 (76%)	3	NA	NA	LR	Recurrence occurred in 38 patients (55%). Five patients developed metastases (6%), four of which had prior RCC in the contralateral kidney.
^A^ Yanagisawa et al. 2019 [93]	2011–2018	18	NA	NA	NA	26	NA	NA	NA	NA	NA	NA	NA	Two patients (11%) developed local recurrence and underwent third ablation.
^A^Vijgh et al. 2017 [64]	2006–2016	20	NA	NA	29	32	NA	NA	NA	NA	NA	NA	LR	Four salvage ablations (20%) were unsuccessful. One patient (5%) developed distant metastases.
Okhunov et al. 2016 [65]	2005–2012	20	NA	14 (70%)	32	30	NA	NA	14 (70%)	2.4	NA	NA	LR	3- and 5-year local RFS was 82%. 5-year OS was 100%. No complications were recorded.
C. Salvage radiotherapy after primary treatment with surgery (*n* = 101)														
Liu et al. 2025 [20]	2021–2025	26	55	NA	NS	22.2	NA	NA	13 (50%)	NA	NA	57.7	LR	2-year local control: 100% 2-year PFS: 91.5%
Liu et al. 2022 [25]	2001–2020	RN = 39	53	26 (67%)	14.6	25.8	NA	NA	25 (64%)	NA	4	43.8***	LR + RR	3-year PFS: 28%, 3- and 5-year OS: 82%
Kim et al. 2021 [54]	2007–2013	PN = 7 RN = 29	NA	23 (64%)	24.1	NA	NA	NA	34 (94%)	NA	NA	NA	LR (58%) RR (42%)	3- and 5-year OS were 71% and 50%, respectively. All recorded deaths were cancer-related.
C. Salvage radiotherapy or ablation after primary treatment with surgery (*n* = 39)														
Porreca et al. 2025 [48]	2015–2024	39	64^W^	73.3^W^	18^W^	62^W^	NA	NA		4.2^W^	NA	NA	LR	3-year LRFS was 72.1%, 3- and 5-year CSS was 98% and 88.1%, respectively 3- and 5-year OS was 95.5% and 80.2%

*AEs *Adverse events, *ccRCC *clear cell renal cell carcinoma, *CSS *Cancer Specific Survival, *DFS *Disease Free Survival, *LR *local recurrence (renal fossa or remaining renal parenchyma), *N *number, *NA *Not Available, *OS *Overall Survival, *PFS *Progression Free Survival, *LRFS *local recurrence-free survival, *RFS *recurrence-free survival, *RR *regional recurrence (retroperitoneal lymph nodes, adrenal gland, or psoas muscles), *RES *residual disease

^#^mean *subset values refer to initial treatment ^A^report published as a conference abstract ^W^data for the whole cohort ** - confirmed biopsies *** - calculated for whole cohort

**Fig. 1:**
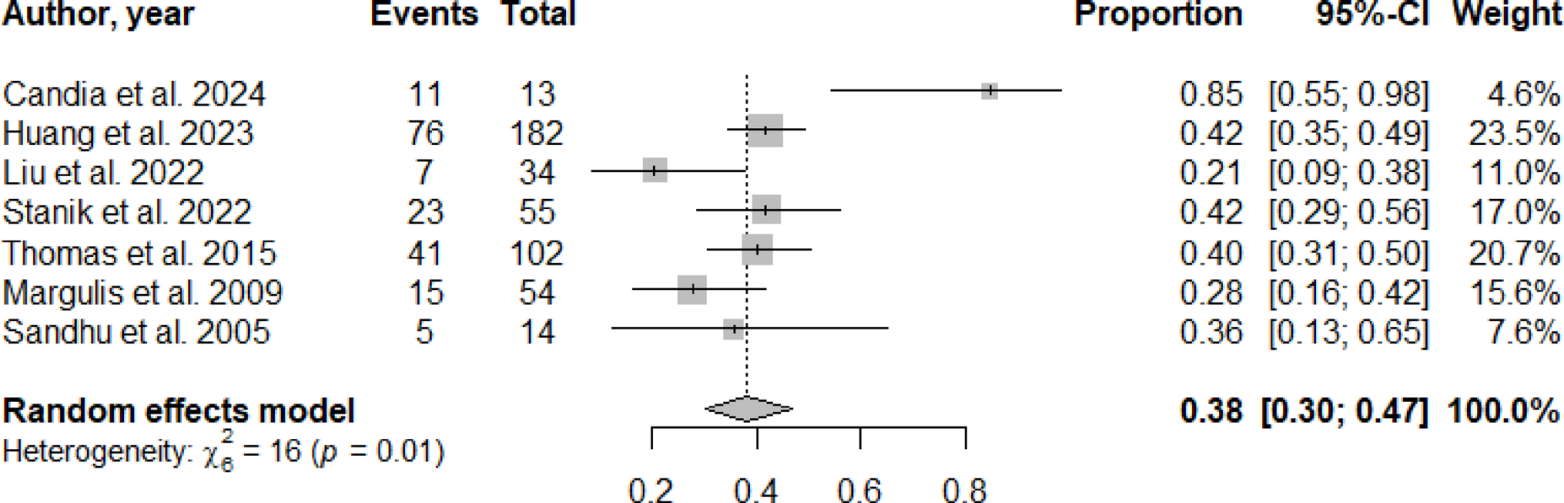
Three-year recurrence-free survival in patients treated with salvage surgery for local recurrence following primary radical nephrectomy

The pooled 3-year RFS of seven studies was 38% (95% CI: 30% to 47%, *n* = 454, Fig. 1), with the majority of studies indicating distant metastases as the primary pattern of failure. There was evidence for significant heterogeneity (*I*^2^ = 62.6%, *p* = 0.01), associated with an outlier study by Candia et al. [22] reporting exceptionally high 3-year RFS of 85%. This small study (*n* = 13) differed from the remainder in every prognostically relevant respect summarised in Table 1: older patients, markedly later recurrences, smaller lesions at salvage, and predominantly T1 disease at primary treatment. A sensitivity analysis excluding study by Candia et al. showed a comparable 3-year pooled RFS of 37% (95% CI: 31% to 43%, *n* = 441), without evidence for significant heterogeneity (*I*^2^ = 38.4%, *p* = 0.15), as presented in Supplementary Fig. 2.

**Fig. 2:**
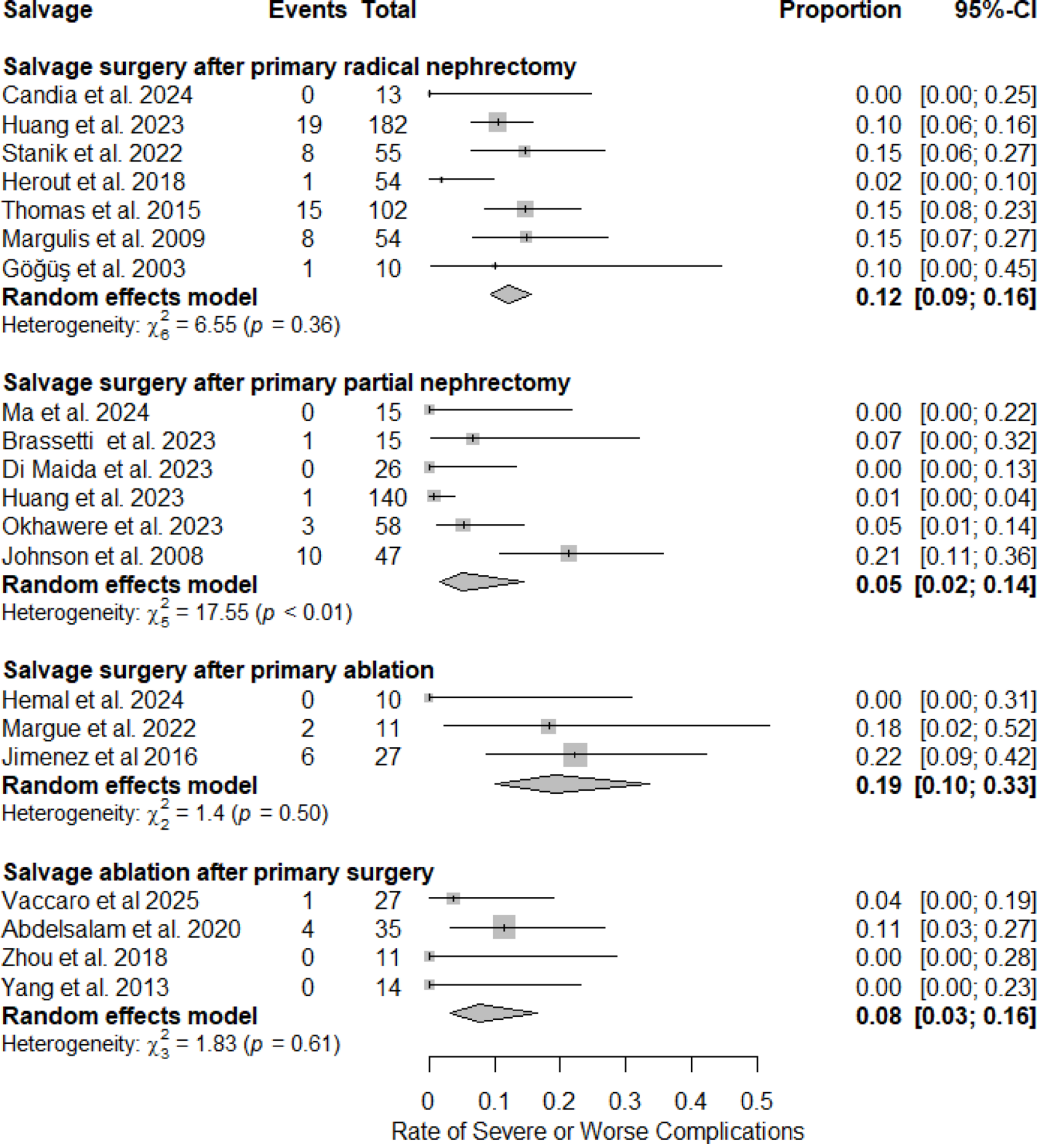
Rates of severe or worse complications across different treatment methods

The pooled 5-year OS and CSS were 62% (95% CI: 55% to 68%, *n* = 522 [Supplementary Fig. 3]) and 61% (95% CI: 53% to 69%, *n* = 417, [Supplementary Fig. 4]), respectively, indicating that vast majority of deaths are attributable to recurrent RCC. An exploratory random-effects meta-regression confirmed a significant positive association between publication year and 5-year OS (permutation *p* = 0.035; k = 9). Predicted 5-year OS rose from approximately 49% in 2008 to 69% in 2023 [Supplementary Fig. 5]. There was no evidence for significant heterogeneity (*I*^2^ = 46.9%, *p* = 0.06 and *I*^2^ = 55.4%, *p* = 0.05, respectively). The pooled rate of severe or worse complications was 12% (95% CI: 9% to 16%, *n* = 470, Fig. 2), with no evidence for significant heterogeneity (*I*^2^ = 8.3%, *p* = 0.4).

### Salvage surgery after primary treatment with partial nephrectomy

Nine studies comprising 410 patients described surgical salvage treatment in patients with local recurrence following partial nephrectomy [19, 21, 33–40] (Table 2A). At primary treatment, tumors were primarily pT1, no patients were reported to have nodal metastases, and the median lesion diameters were relatively low, ranging from 3 to 4.5 cm. At salvage treatment, all of the studies included patients with local renal recurrence, and the median lesion size ranged from 2 to 3.5 cm. RoB analysis revealed six studies had moderate RoB, mainly because of bias in participant selection and outcome measurement [19, 33–36, 40]. The remaining three studies were assessed as serious RoB, two were published as abstracts [37, 38], and one included only patients with VHL syndrome [39].

The anatomical definition of “local recurrence” differed across included studies and primarily was restricted to intrarenal recurrences. Studies by Brassier et al. and Huang et al. excluded recurrences in ipsilateral renal fossa, adrenal gland and retroperitoneal lymph nodes (for Brassier et al. additionally metachronous lesions in the ipsilateral kidney away from original partial nephrectomy bed were excluded) [19, 36]. In reports by Di Maida et al. and Antonelli et al. recurrences in resection bed and elsewhere in ipsilateral kidney were distinguished (53.8% vs. 46.2% and 53% vs. 47%, respectively), but did not include loco-regional recurrences [35, 38]. Ma et al. allowed for retroperitoneal recurrences; however, no such cases were reported (86.7% resection bed, 13.3% ipsilateral kidney) [33]. The anatomical site of recurrence was not specified in remaining studies. In the most recent series, Zhou et al. divided 55 ipsilateral recurrences as occurring either within the original resection bed (58.2%) or in ipsilateral parenchyma remote from it (41.8%), and found they differ pathologically: upstaging at salvage occurred in 84.3% of original-site compared with 21.7% of non-original-site recurrences (*p* < 0.001) [40].

Only one study (*n* = 140; >90% of patients with T1 disease) reported RFS as time-to-event endpoint, with 3- and 5-year estimations of 78% and 76%, respectively. The outcomes were heterogeneous, ranging from treatment failures primarily consisting of distant metastases, exceeding half of the cases [19, 38], to studies reporting as few as 7% to 19% recurrences [33, 35, 37]. Okhawere et al. reported promising outcomes (3–5% recurrence rates) in a cohort of patients including those with pT3/pT4 pathologic stage; however, the study had very short median follow-up (7 months) [34]. In a study by Di Maida et al. (77% primarily pT1 disease) local and systemic recurrences occurred in 11.5% and 7.7% of patients, respectively [35]. Finally, Johnson et al. reported outcomes of salvage partial nephrectomy in patients with von Hippel-Lindau syndrome (VHL), showing local recurrences in 20% of the operated kidneys, and distant metastases in 6% of the patients at a median follow-up of 56 months [39]. The majority of studies reported none or few cancer-related deaths. Zhou et al. reported secondary recurrence in 8 of 55 patients (14.5%), progression to metastatic disease in 7.3%, and cancer-specific death in 10.9%, with no significant difference in recurrence-free or overall survival between the two recurrence patterns (*p* = 0.31 and *p* = 0.16); salvage radical nephrectomy was required in 81.2% of original-site compared with 47.8% of non-original-site recurrences (*p* = 0.003) [40].

Severe or worse complications occurred in 5% (95% CI: 2% to 14%, *n* = 301, Fig. 2), with evidence for significant heterogeneity (*I*^2^ = 71.5%, *p* < 0.01). Five of the six studies included in the analysis were published between 2023 and 2024, while the study by Johnson et al., reporting high rates of complications (21%), was published in 2008 [39]. After excluding the study by Johnson et al., the pooled rate of complications was 3% (95% CI: 1% to 8%, *n* = 254, [Supplementary Fig. 6]), with no evidence for significant heterogeneity (*I*^2^ = 0%, *p* = 0.4).

### Salvage surgery after primary treatment with ablation

Three studies including 48 patients described outcomes of salvage surgical treatments for recurrence following ablative therapies [41–43] (Table 2B). Most patients were treated with salvage partial nephrectomy. The median lesion sizes at salvage ranged from 2.3 to 3.6 cm, and none of the studies included patients with regional recurrences. Limited details were provided regarding patient characteristics at primary treatment. None of the three studies reported pTNM staging or tumor grade at primary ablation, and histological subtype was inconsistently reported (primarily clear cell RCC). Two studies were assessed as moderate RoB, mainly because of bias in participant selection [41, 42], one study was judged to be serious RoB, because in relatively small cohort of 27 patients seven were lost to follow-up [43]. At relatively short median follow-up periods, ranging from 12 to 16 months, the authors reported local recurrence rates of 4% to 10% and 0% to 18% distant failure rates. The pooled rate of severe or worse complications was 19% (95% CI: 10% to 33%; *n* = 48; Fig. 2), with no evidence for significant heterogeneity (*I*^2^ = 0%, *p* = 0.5).

### Salvage surgery after heterogeneous initial treatments

Eleven studies including 543 patients described outcomes of salvage surgery in patients previously treated with heterogeneous approaches, including radical nephrectomy, partial nephrectomy, and ablative therapies, and patients treated for both local and regional recurrences [44–54] (Table 2C). In this group, pathological stages were also heterogeneous: studies including more patients primarily treated with ablation generally involved less advanced disease, whereas studies with higher rates of radical nephrectomy included patients with more advanced pathological stages. One study (*n* = 19) focused on patients with local lymph node metastases treated with salvage lymphadenectomy [48], and another study (*n* = 12) reported outcomes of surgery combined with intraoperative and adjuvant radiotherapy [53]. Three studies were assessed as having serious RoB: two were available only as abstracts [47, 53], and the study by Porreca et al. did not report salvage treatment outcomes separately according to the primary treatment modality. Additionally, its multicentre design may have introduced bias in participant selection and outcome reporting [44].

Studies reporting outcomes of salvage treatment for local recurrences following partial nephrectomy or ablation reported distant failure rates up to 15%, and few or no local recurrences [46, 47, 52]. On the contrary, studies including patients previously treated with radical nephrectomy and/or treated for regional recurrences presented with significantly higher recurrence rates, reaching up to 70% at five years, and estimated 25% to 39% cancer-specific mortality at five years [48, 50, 51]. One study (*n* = 155) reported relatively high 5-year CSS of 94% despite inclusion of primarily post-radical nephrectomy patients, and patients with regional recurrences [54, 68]. Rates of complications were heterogeneous, likely reflecting differences between studies and cohorts, as summarized in Table 2C. In RoB analysis eight studies were judged to have moderate RoB, mainly because of bias in participant selection and outcome measurement [45, 46, 48–52, 54].

### Salvage ablation after primary treatment with partial nephrectomy

Six studies including 152 patients described outcomes of salvage ablation after partial nephrectomy (Table 3A) [19, 55–59]. One study also included three patients previously treated with radical nephrectomy [57]. These studies included only patients with local recurrences, with a median lesion size ranging from 2.1 to 2.8 cm, except for the study by Krajewski et al. which also included patients with residual disease and regional recurrences [59]. Few details were reported regarding disease stage at primary treatment. Primary pathological tumor stage was reported only in the study by Brassier et al., in which 70% of patients had pT1 disease [19]. Three studies were assessed to have moderate RoB [19, 55, 57], and three studies as serious RoB: the first was published only as abstract [56], the second did not clearly define selection criteria and had concerns regarding outcomes reporting [59], and the third included only patients with VHL disease, non-representative of general population [58].

The definition of “local recurrence” in the post-PN ablation studies was almost uniformly limited to intrarenal disease, similar to the surgical PN-salvage subgroup. Brassier et al. excluded loco-regional recurrences [19], and Abdelsalam et al. and Vaccaro et al. restricted inclusion to recurrences in the ipsilateral kidney after PN [55, 56]. Yang et al. enrolled patients with VHL syndrome whose recurrences were intrarenal and multifocal [58]. A study by Zhou et al. (2018) was the only that explicitly included ipsilateral loco-regional disease, and in eight patients treated primarily with partial nephrectomy seven recurrences were in renal fossa [57] and in study by Krajewski et al. 12 patients had locoregional recurrence; however, the exact localization was not specified [59].

**Fig. 3:**
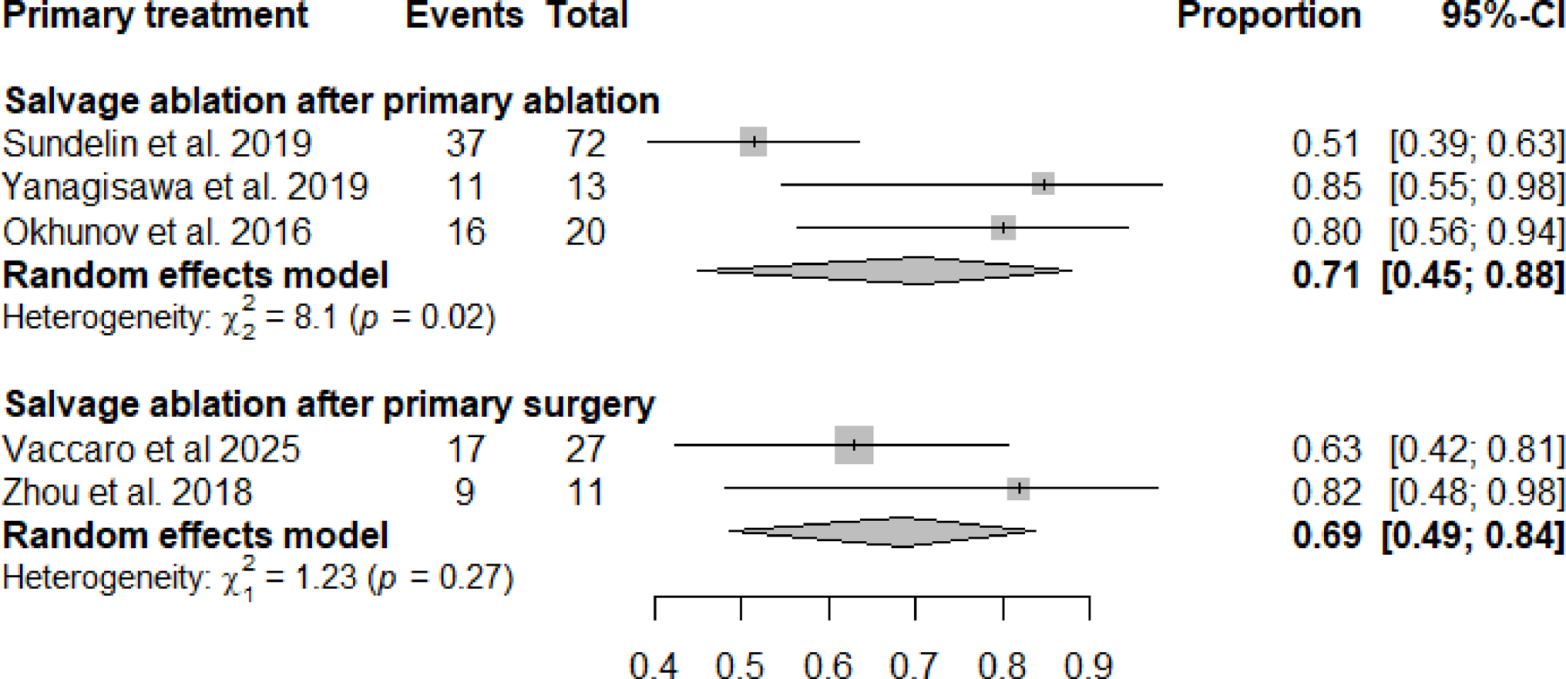
Three-year recurrence-free survival in patients treated with salvage ablation

The pooled 3-year RFS was 69% (95% CI: 49% to 84%, Fig. 3) across two studies (*n* = 38), with no evidence for significant heterogeneity (*I*^2^ = 18.7%, *p* = 0.3) [55, 57]. One study (*n* = 14) included only patients with VHL, reporting four (29%) local recurrences during follow-up [58]. The studies by Abdelsalam et al. and Brassier et al. reported local recurrence rates of 6% and 19%, with 0% and 10% distant failure rates, respectively [19, 56]. The study by Krajewski et al. reported two non-cancer-related deaths during follow-up and no metastatic spread [59]. The rate of severe complications was 8% (95% CI: 3% to 16%), with no evidence for significant heterogeneity (*I*^2^ = 0%, *p* = 0.6).

### Salvage ablation after primary treatment with ablation

Six studies including 193 patients described outcomes of salvage ablation after primary ablation failure [60–65]. Few details were provided regarding cohort characteristics at primary or salvage treatment, except for median lesion size at primary ablation, which ranged between 2.4 and 3.7 cm in four studies. RoB analysis showed three studies to have moderate RoB [61, 62, 65] and three studies published only as abstracts without clearly defined inclusion criteria were assessed to have serious RoB [60, 63, 64].

The pooled 3-year RFS was 71% (95% CI: 45% to 88%, *n* = 105, Fig. 3) with evidence for significant heterogeneity (*I*^2^ = 75.3%, *p* = 0.02) attributable to the study by Sundelin et al. However, no qualitative differences justifying a sensitivity analysis were identified. The remaining three studies reported 8% to 25% rates of failure, primarily attributable to local recurrences [60, 61, 64]. One study (*n* = 20) reported no severe complications [65].

### Salvage radiotherapy after primary treatment with surgery

Three studies including 101 patients described outcomes of salvage radiotherapy after primary radical nephrectomy (*n* = 68) or partial nephrectomy (*n* = 7) [25, 54], including patients treated for both local and regional recurrences. No details regarding the initial stage of the disease were provided. The prospective study by Liu et al. did not report type of initial surgical treatment (*n* = 26) [20]. All three studies were assessed as having moderate RoB.

The retrospective study by Liu et al. (*n* = 39) reported 3-year PFS of 28% and 5-year OS of 82%, while Kim et al. (*n* = 36) reported 5-year CSS of 50%. In a prospective study comprising patients with locoregional recurrences by Liu et al., in which radiotherapy was administered together with systemic therapy, the 2-year local control rate was 100% and 2-year PFS was 91.5%; however AEs grade ≥ 3 were substantial, observed in 50% of participants, most frequently hypertension and leukopenia [20].

### Salvage radiotherapy or ablation after primary treatment with surgery

One study evaluated the efficacy of radiotherapy or ablation after primary surgical treatment (*n* = 39) [44]. The study was assessed as serious risk of bias, because of retrospective multicenter registry design without clearly defined inclusion criteria. The 3-year local control rate was 72.1%, while the 5-year CSS and OS were 92.7% and 80.2%, respectively.

### Comparative studies

Five studies including 590 patients directly compared outcomes of different salvage treatment strategies [19, 25, 36, 44, 54]. Due to heterogeneity in study design, patient populations, comparators and outcome definitions, meta-analysis was omitted.

For surgical methods, Huang et al. compared outcomes of salvage partial (*n* = 34) and radical (*n* = 106) nephrectomy [36]. After a median follow-up of 27 months there were no differences in local recurrence-free survival (HR 1.08; *p* > 0.9) and RFS (HR = 0.74; *p* = 0.6), but after longer follow-up partial nephrectomy showed superiority in renal function preservation with a mean eGFR of 71.4 vs. 54.0 ml/min/1.73 m² (*p* < 0.001) and a mean eGFR loss of 6.6% vs. 25.6% (*p* < 0.001) after matching.

Brassier et al. [19] compared ablation with salvage surgery. There were no differences in risk of disease recurrence (HR = 0.72; *p* = 0.6), local control (HR = 1.51; *p* = 0.6) or distant metastases (HR = 0.19; *p* = 0.09). However, ablation was associated with lower risk of postoperative complications (5% vs. 41%; odds ratio = 0.22; *p* = 0.006) and better renal function preservation (− 16.18 ml/min/1.73 m^2^; *p* = 0.001).

Local therapy was also compared with systemic treatment in a study by Liu et al. [25]. Patients receiving local therapy (*n* = 73; surgery *n* = 34, SBRT *n* = 39) were compared with those receiving systemic therapy alone (*n* = 33), using propensity score matching (*n* = 31 per arm). At a median follow-up of 25.8 months, local therapy was associated with significantly longer median PFS (23.9 vs. 7.5 months, *p* = 0.001), but no significant difference in 2-year OS (91.6% vs. 71.8%, *p* = 0.084). Among patients receiving local therapy, no significant difference was found between surgery and SBRT in terms of PFS (*p* = 0.523) or OS (*p* = 0.312), while 1-year local control was numerically higher with SBRT (96.6% vs. 75.1%).

Comparisons were also performed using within multi-institutional registries. Porreca et al. compared surgical resection to ablation/radiotherapy in patients treated in an Italian registry, and showed significantly improved 3-year local control after surgical treatment (86.4% vs. 72.1%; *p* = 0.02); however, there was no statistical difference in terms of 5-year CSS (92.7% vs. 88.1%; *p* = 0.18) [44]. In a Korean registry by Kim et al., secondary treatments consisted of surgery, targeted therapy, other systemic therapy, or radiotherapy [54]. Median OS was longest in patients undergoing surgery (54.9 months), compared to targeted therapy (41.7 months), systemic therapy (42.9 months), and radiotherapy (38.0 months; *p* < 0.001). These findings should be interpreted in the context of the study’s limitations, including patient selection, absence of radiological staging data, and lack of site-specific recurrence information.

## Discussion

In this systematic review and meta-analysis of 47 studies involving 2039 patients, we comprehensively evaluated the effectiveness of local salvage therapies for RCC recurrence stratified by the initial treatment modality. Our findings reveal that outcomes are highly heterogeneous and strongly dependent on the prior treatment received and the advancement of the primary disease. Repeat ablation demonstrated favorable outcomes after primary ablation, with pooled 3-year RFS of 71% (95% CI: 45%–88%), while salvage surgery showed promising but less robust results. For recurrences following partial nephrectomy, repeat surgery was associated with a 3-year RFS of 78%, though outcomes were heterogeneous ranging from low recurrence rates (7–19%) to distant metastatic failure in over half of the patients. There was also evidence supporting the use of salvage ablation. In contrast, surgical salvage after radical nephrectomy was associated with a pooled 3-year RFS of 38% (95% CI: 30%–47%), driven primarily by distant metastatic progression. Very limited evidence supported alternative approaches in this scenario, like ablation or radiotherapy.

We found that repeat ablation (radiofrequency or cryoablation) represents an effective strategy following primary ablation failure. The pooled 3-year RFS of 71% (95% CI: 45%–88%) and 5-year RFS of 61% (95% CI: 23%−89%) suggest that many patients can benefit from this approach. However, a considerable variation in outcomes, which may be due to differences in tumor characteristics or the exact methods used for the procedure. Additionally, this comparison included mainly small studies, often published only as conference abstracts. Importantly, TNM staging at primary treatment was not reported in any of the studies what limits the ability to assess the impact of primary tumor biology on salvage outcomes in this group.

Three studies also assessed role of salvage surgery in post-ablation setting, but they were mainly small series without long-term follow-up data. The pooled severe complication rate of 19% is higher than observed in other subgroups, which may reflect the technical challenges of operating in previously ablated tissue with perinephric fibrosis, and distorted anatomy [43, 66]. Given the overall small number of patients and short follow-up, these findings should be interpreted with caution; however, they indicate that post-ablation salvage surgery is feasible and may offer cure in carefully selected patients. Also, in this setting no TNM staging for primary or recurrent disease was reported.

In patients with recurrence after partial nephrectomy, both repeat surgery and ablation show promising results, though outcomes across included studies were heterogeneous. Salvage surgery encompassing partial or radical nephrectomy was performed across eight studies, with one large study (*n* = 140) reporting 3- and 5-year RFS of 78% and 76% respectively. These relatively favorable results may reflect the small tumor sizes at salvage (median 2–2.6 cm) and the absence of regional recurrences. Notably, tumors at primary treatment in this subgroup were predominantly staged as pT1 with no reported nodal metastases, suggesting a more favorable underlying tumor biology compared with the post-radical nephrectomy population. The decision between repeat partial versus radical nephrectomy remains clinically challenging. In a comparative study by Huang et al. there were no differences in local control or RFS, while partial nephrectomy significantly better-preserved renal function. These findings support the use of repeat partial nephrectomy where technically feasible, consistent with the nephron-sparing recommendation in primary treatment. In same group of patients, salvage ablation was evaluated in five studies, with a local recurrences rate of 8–29%, suggesting a potentially inferior local control rate compared to repeat surgery. However, in a study directly comparing these methods there were no differences in recurrence risk, local control, or distant metastasis rates. Ablation was superior in terms of renal preservation and risk of postoperative complications, but these results are biased, because of retrospective, observational design of a study [19]. The choice between ablation and surgery in this setting should therefore be individualized based on patient comorbidities, renal function status, tumor location and size, and expected operative complexity – ideally within interdisciplinary team. Importantly, recurrence after partial nephrectomy may represent a biologically distinct disease. Loco-regional recurrence sites, such as the renal fossa, adrenal gland, retroperitoneal lymph nodes, and peritoneum, were underreported in the included studies. In the only study including a substantial proportion of renal fossa recurrences, by Zhou et al. (2018), the 3-year RFS was favorable (82%) [57]. In multicenter retrospective analysis of atypical locoregional peritoneal recurrence after nephrectomy for localized RCC, isolated peritoneal recurrence was significantly more frequent after partial nephrectomy than after radical nephrectomy (OR 4.1, 95% CI 1.7–9.5) [67]. However, the data were insufficient for a dedicated site-specific analysis involving renal fossa, regional lymph nodes, adrenal gland, or psoas muscles, and peritoneal recurrences were not the focus of our analysis. Within the ipsilateral kidney, however, recurrence site does appear to carry biological meaning. Zhou et al. reported that recurrences arising in the original resection bed were upstaged at salvage in 84.3% of cases, compared with 21.7% of those arising remote from the bed, and required salvage radical nephrectomy more often (81.2% vs. 47.8%), while repeat partial nephrectomy predominated at non-original sites (47.8% vs. 9.4%); cancer-specific mortality was numerically but not significantly higher after original-site recurrence [40]. It supports evidence that non-original-site lesions may reflect multifocal or de novo tumor genesis rather than regrowth of incompletely treated disease, and that recurrence location may help select patients for repeat nephron-sparing surgery.

An important consideration are patients with VHL syndrome, Johnson et al. reported 20% local recurrences per operated kidney in VHL patients undergoing salvage partial nephrectomy, with low rates of cancer-related mortality at extended follow-up, reflecting the indolent biology of VHL-associated RCC [39]. These patients require distinct management strategies and should be treated at specialized centers. An option for them could be use of SBRT [68]. An important caveat is that, in patients with VHL syndrome, metachronous ipsilateral lesions may represent de novo primary tumors arising from the germline predisposition rather than true recurrences of the originally treated tumor. The included studies did not distinguish between these possibilities, and Johnson et al. reported events per operated kidney rather than per lesion of origin, so the apparent local recurrence rates in this subgroup probably overestimate genuine local treatment failure [39]. Both VHL-restricted studies were rated at serious RoB, and neither contributed to a pooled recurrence-free survival estimate.

Patients recurring after radical nephrectomy were at highest risk of failure, predominantly due to distant metastatic progression occurring in 42–55% of patients, highlighting the difficulties in managing recurrent RCC after a radical nephrectomy. This is consistent with clinically advanced disease at primary treatment (pT3–4 in 27–69% of patients) and frequent nodal involvement, likely leading to high risk of concomitant subclinical metastatic disease at local recurrence. However, with the recent introduction of adjuvant systemic approaches potentially reducing the impact of subclinical distant disease in intermediate- or high-risk RCC, these results should be re-evaluated [69]–particularly in context of pembrolizumab, which showed OS benefit in patients with intermediate to high-risk RCC [70]. Supporting this, an exploratory meta-regression of 5-year OS by publication year revealed a significant positive association, corresponding to a predicted increase in 5-year OS from approximately 49% in 2008 to 69% in 2023. Several prognostic factors emerged as relevant across the included studies. Later time to recurrence, smaller lesion size at salvage, and absence of regional recurrence were consistently associated with more favorable outcomes, while locally advanced primary disease (pT3–4), nodal involvement at initial surgery, and large recurrence size were associated with higher failure rates. Further evidence supporting definitive local treatment comes from prospective RECUR registry, that showed significant survival benefit across all RCC-recurrence groups, even those with risk of subclinical systemic disease [71].

These stage-related differences between subgroups are important to read our results correctly. The more favorable outcomes seen after salvage of post-ablation and post-partial-nephrectomy recurrences, compared with post-radical-nephrectomy recurrences, do not indicate that one salvage modality outperforms another. They reflect confounding by indication: the primary treatment is itself selected on the basis of tumor stage, size, and biology, so patients initially treated with radical nephrectomy begin from locally advanced, frequently high-grade disease with a higher baseline risk of both local and distant failure, while patients managed with ablation or partial nephrectomy are typically those with small, low-stage tumors. For this reason, we stratified every analysis by primary treatment modality, made no statistical comparison across subgroups, and present pooled estimates as descriptive summaries of what may be expected within each clinical scenario rather than as estimates of comparative effectiveness. Considered with this caution, the stratified estimates retain clinical value: by the time a patient presents with a local recurrence, the primary treatment they received is already known, and these data describe the oncological outcomes and complication rates that can be anticipated in that situation.

The evidence for the role of ablative treatment - SBRT in the post-radical nephrectomy setting remains limited. Salvage radiotherapy was evaluated in two retrospective studies with limited dosing details and one small prospective study combining SBRT with sintilimab and axitinib, none of which provided details on clinical disease stage at primary treatment. The latter study showed improved results compared to the radiotherapy alone, but with high rate of grade ≥ 3 AEs of 50%, indicating that toxicity management will be a problem for combination treatment approaches. SBRT alone is a non-invasive option with the potential for favorable local control in the renal fossa, as supported by emerging data in the primary and oligometastatic settings [10, 72]. The phase II FASTRACKII study evaluating the use of SBRT in 71 patients with primary RCC showed 100% local control rate at 84 months, and pooled analysis with phase I FASTTRACK showed local control rate at five years of 98% with progression in only one patient [73, 74]. Excellent results were confirmed in IROCK individual-patient-data meta-analysis showing 5-years local failure rate of 5.5% [75], and the ISRS practice guideline provided a consensus framework for dose-fractionation (26 Gy×1, 42 Gy×3) [76]. In oligometastatic RCC SBRT a study by Franzese et al. showed good efficacy in controlling oligorecurrent and oligoprogressive RCC, with favorable safety profile also when combined with systemic therapy [77]. However, within salvage population the propensity-matched comparison of SBRT and surgery groups found no significant difference in PFS and OS between groups, while 1-year local control was numerically higher in SBRT group [25]. Dedicated studies in the salvage post-RN setting are lacking and prospective registries assessing the role of SBRT in post-nephrectomy local recurrence are needed.

Across all salvage treatment modalities, rates of severe complications ranged from 3 to 19%, with the highest complication rate in salvage surgery after ablation, reflecting the technical complexity of operating in previously ablated tissue. In the largest comparative study by Brassier et al. [19] on post-surgical recurrence management, ablation was associated with significantly lower complication rate than surgery (5% vs. 41%; OR 0.22, *p* = 0.006), suggesting the benefit of using ablation in patients with impaired renal function or comorbidities. These complication rates should be considered in the broader context of potential benefit, as effective salvage of a local RCC recurrence may delay or eliminate the need for systemic therapy with its associated chronic toxicity burden.

Main limitations of our study include the predominantly retrospective design of included trials, only one prospective registry and one single-arm study, lack of randomized controlled trials resulting in overall moderate quality of evidence according to RoB. Selection of participants is the main bias criterion, as patients eligible for aggressive local salvage therapy likely represent a highly selected, healthier subpopulation compared to those managed conservatively. Second, significant heterogeneity was present across studies in patient characteristics, recurrence definitions, salvage treatment techniques, outcome definitions, and follow-up durations, limiting the interpretability of pooled estimates. Additionally, our pooling of Kaplan–Meier-derived event probabilities at fixed timepoints approach only approximates time-to-event data and assuming similar censoring across studies. Third, many studies, particularly in the ablation subgroups, were published as conference abstracts with limited methodological detail, hindering thorough quality assessment. Pre-specified threshold (≥ 10 studies per analysis) was not met and no publication-bias analysis was performed. Fourth, rapid evolution of systemic therapies, including adjuvant immunotherapy, may alter the oncological context in which local salvage therapies are used. Additionally, the quality and quantity of evidence on this topic is limited, making it extremely challenging to draw definitive conclusions on which technique is most appropriate to treat recurrence in specific patient groups based on available studies. It is consistent with the ongoing debate between ablation or nephron-sparing surgery for localized renal masses, as robust quality indicators for RCC care and their association with clinical outcomes remain insufficiently defined [78, 79]. Therefore, there is critical need to establish expert consensus on quality indicators for locally recurrent RCC. Developing such indicators is substantial for optimal design of prospective multicenter registries with standardized outcome reporting, which will form backbone of future studies aimed at optimizing management of patients with recurrent RCC. Moreover, future research priorities are trials with standardized patient selection, treatment details, as well as studies integrating biomarkers and molecular profiling to identify patients most likely to benefit from local versus systemic approaches. Fifth, although the protocol permitted inclusion of isolated recurrences in the ipsilateral adrenal gland, retroperitoneal lymph nodes, and psoas muscle, no included study reported outcomes separately for these sites and no cohort of isolated adrenal recurrence was identifiable; site-specific estimates outside the kidney and renal fossa could therefore not be derived, and the boundary between loco-regional recurrence and oligometastatic disease at these sites remains a recognized source of classification uncertainty. Finally, and most importantly, the differences observed between subgroups are confounded by indication and must not be interpreted as comparisons between salvage modalities.

## Conclusion

The effectiveness of treatments for recurrent RCC varies depending on primary treatment modality and the underlying disease biology. Repeat ablation provides favorable oncological outcomes. Recurrence after partial nephrectomy can be managed with both repeat surgery and ablation; however, outcomes are heterogeneous, ranging from low recurrence rates to frequent distant metastases. Patients recurring after radical nephrectomy, are usually initially treated for more advanced disease. Majority experience disease progression within three years, primarily due to distant metastases, underscoring possible necessity of integrating systemic treatment. Non-invasive ablative approaches, such as SBRT, are emerging.

Overall, the quality and quantity of evidence remains low, and is primarily retrospective. Salvage treatment of local RCC recurrence is a complex clinical scenario that requires multidisciplinary team. There is a critical need for expert consensus on quality indicators for locally recurrent RCC care and for prospective multicenter registries with standardized outcome reporting to optimize management for this challenging patient population.

## Supplementary Information

Below is the link to the electronic supplementary material.

Supplementary Material 1

## Data Availability

No datasets were generated or analysed during the current study.
