# Supplementary material for: Salvage therapies for local recurrence of renal cell carcinoma: a systematic review and meta-analysis
Source: World J Urol. 2026 Aug 16;44(1):568. doi: 10.1007/s00345-026-06684-8 (PMC13478036; doi:10.1007/s00345-026-06684-8)
Supplement: Supplementary file 1 [file 345_2026_6684_MOESM1_ESM.docx]

**Supplementary Information:**

1. **Supplementary Table 1.** PRISMA 2020 for Abstracts Checklist
2. **Supplementary Table 2.** PRISMA 2020
3. **Supplementary Table 3.** PICOS
4. **Supplementary Table 4.** Risk Of Bias In Non-randomized Studies - of Interventions (ROBINS I)
5. Supplementary Figure 1. PRISMA flow diagram
6. Supplementary Figure 2. Sensitivity Analysis for Three-Year Recurrence-Free Survival in Patients Treated With Salvage Surgery for Local Recurrence Following Primary Radical Nephrectomy
7. Supplementary Figure 3. Five-Year Overall Survival in Patients Treated With Salvage Surgery for Local Recurrence Following Primary Radical Nephrectomy
8. Supplementary Figure 4. Five-Year Cancer-Specific Survival in Patients Treated With Salvage Surgery for Local Recurrence Following Primary Radical Nephrectomy
9. Supplementary Figure 5. Meta-regression of 5-year overall survival by publication year in patients treated with salvage surgery for local recurrence following radical nephrectomy
10. Supplementary Figure 6. Sensitivity Analysis for Rate of Severe or Worse Complications in Patients	Treated With Salvage Surgery for Local Recurrence Following Primary Partial Nephrectomy
11. **Supplementary Appendix 1**. AMSTAR-2 checklist
12. **Supplementary Appendix 2.** Search strategy
13. **References**

**Abbreviations:**

AMSTAR-2 = A Measurement Tool to Assess Systematic Reviews 2, PRISMA = Preferred Reporting Items for Systematic Reviews and Meta-Analyses, ROBINS-I = Risk Of Bias In Non-randomized Studies - of Interventions. Rob 2= A revised Cochrane Risk-of-Bias tool for randomized trials

**Supplementary Table 1.** PRISMA 2020 for Abstracts Checklist

| **Section and Topic** | **Item #** | **Checklist item** | **Reported (Yes/No)** |
| --- | --- | --- | --- |
| **TITLE** | | |  |
| Title | 1 | Identify the report as a systematic review. | Yes |
| **BACKGROUND** | | |  |
| Objectives | 2 | Provide an explicit statement of the main objective(s) or question(s) the review addresses. | Yes |
| **METHODS** | | |  |
| Eligibility criteria | 3 | Specify the inclusion and exclusion criteria for the review. | Yes |
| Information sources | 4 | Specify the information sources (e.g. databases, registers) used to identify studies and the date when each was last searched. | Yes |
| Risk of bias | 5 | Specify the methods used to assess risk of bias in the included studies. | Yes |
| Synthesis of results | 6 | Specify the methods used to present and synthesise results. | Yes |
| **RESULTS** | | |  |
| Included studies | 7 | Give the total number of included studies and participants and summarise relevant characteristics of studies. | Yes |
| Synthesis of results | 8 | Present results for main outcomes, preferably indicating the number of included studies and participants for each. If meta-analysis was done, report the summary estimate and confidence/credible interval. If comparing groups, indicate the direction of the effect (i.e. which group is favoured). | Yes |
| **DISCUSSION** | | |  |
| Limitations of evidence | 9 | Provide a brief summary of the limitations of the evidence included in the review (e.g. study risk of bias, inconsistency and imprecision). | Yes |
| Interpretation | 10 | Provide a general interpretation of the results and important implications. | Yes |
| **OTHER** | | |  |
| Funding | 11 | Specify the primary source of funding for the review. | Yes |
| Registration | 12 | Provide the register name and registration number. | Yes |

**Supplementary Table 2.** PRISMA 2020

| **Section and Topic** | **Item #** | **Checklist item** | **Location where item is reported** |
| --- | --- | --- | --- |
| **TITLE** | | |  |
| Title | 1 | Identify the report as a systematic review. | Page1 |
| **ABSTRACT** | | |  |
| Abstract | 2 | See the PRISMA 2020 for Abstracts checklist. | Page4 |
| **INTRODUCTION** | | |  |
| Rationale | 3 | Describe the rationale for the review in the context of existing knowledge. | Page5 |
| Objectives | 4 | Provide an explicit statement of the objective(s) or question(s) the review addresses. | Page5 |
| **METHODS** | | |  |
| Eligibility criteria | 5 | Specify the inclusion and exclusion criteria for the review and how studies were grouped for the syntheses. | Page6 |
| Information sources | 6 | Specify all databases, registers, websites, organisations, reference lists and other sources searched or consulted to identify studies. Specify the date when each source was last searched or consulted. | Page6 |
| Search strategy | 7 | Present the full search strategies for all databases, registers and websites, including any filters and limits used. | Supplementary |
| Selection process | 8 | Specify the methods used to decide whether a study met the inclusion criteria of the review, including how many reviewers screened each record and each report retrieved, whether they worked independently, and if applicable, details of automation tools used in the process. | Page6-7 |
| Data collection process | 9 | Specify the methods used to collect data from reports, including how many reviewers collected data from each report, whether they worked independently, any processes for obtaining or confirming data from study investigators, and if applicable, details of automation tools used in the process. | Page6-7 |
| Data items | 10a | List and define all outcomes for which data were sought. Specify whether all results that were compatible with each outcome domain in each study were sought (e.g. for all measures, time points, analyses), and if not, the methods used to decide which results to collect. | Page6-7 |
|  | 10b | List and define all other variables for which data were sought (e.g. participant and intervention characteristics, funding sources). Describe any assumptions made about any missing or unclear information. | Page6-7 |
| Study risk of bias assessment | 11 | Specify the methods used to assess risk of bias in the included studies, including details of the tool(s) used, how many reviewers assessed each study and whether they worked independently, and if applicable, details of automation tools used in the process. | Page7 |
| Effect measures | 12 | Specify for each outcome the effect measure(s) (e.g. risk ratio, mean difference) used in the synthesis or presentation of results. | Page7 |
| Synthesis methods | 13a | Describe the processes used to decide which studies were eligible for each synthesis (e.g. tabulating the study intervention characteristics and comparing against the planned groups for each synthesis (item #5)). | Page7 |
|  | 13b | Describe any methods required to prepare the data for presentation or synthesis, such as handling of missing summary statistics, or data conversions. | Page7 |
|  | 13c | Describe any methods used to tabulate or visually display results of individual studies and syntheses. | Page7 |
|  | 13d | Describe any methods used to synthesize results and provide a rationale for the choice(s). If meta-analysis was performed, describe the model(s), method(s) to identify the presence and extent of statistical heterogeneity, and software package(s) used. | Page7 |
|  | 13e | Describe any methods used to explore possible causes of heterogeneity among study results (e.g. subgroup analysis, meta-regression). | Page7 |
|  | 13f | Describe any sensitivity analyses conducted to assess robustness of the synthesized results. | Page7 |
| Reporting bias assessment | 14 | Describe any methods used to assess risk of bias due to missing results in a synthesis (arising from reporting biases). | Page7 |
| Certainty assessment | 15 | Describe any methods used to assess certainty (or confidence) in the body of evidence for an outcome. | Page7 |
| **RESULTS** | | |  |
| Study selection | 16a | Describe the results of the search and selection process, from the number of records identified in the search to the number of studies included in the review, ideally using a flow diagram. | Page8-10 |
|  | 16b | Cite studies that might appear to meet the inclusion criteria, but which were excluded, and explain why they were excluded. | Page8-10 |
| Study characteristics | 17 | Cite each included study and present its characteristics. | Page8-10 |
| Risk of bias in studies | 18 | Present assessments of risk of bias for each included study. | Page8-10 |
| Results of individual studies | 19 | For all outcomes, present, for each study: (a) summary statistics for each group (where appropriate) and (b) an effect estimate and its precision (e.g. confidence/credible interval), ideally using structured tables or plots. | Page8-10 |
| Results of syntheses | 20a | For each synthesis, briefly summarise the characteristics and risk of bias among contributing studies. | Page8-10 |
|  | 20b | Present results of all statistical syntheses conducted. If meta-analysis was done, present for each the summary estimate and its precision (e.g. confidence/credible interval) and measures of statistical heterogeneity. If comparing groups, describe the direction of the effect. | Page8-10 |
|  | 20c | Present results of all investigations of possible causes of heterogeneity among study results. | Page8-10 |
|  | 20d | Present results of all sensitivity analyses conducted to assess the robustness of the synthesized results. | Page8-10 |
| Reporting biases | 21 | Present assessments of risk of bias due to missing results (arising from reporting biases) for each synthesis assessed. | Page8-10 |
| Certainty of evidence | 22 | Present assessments of certainty (or confidence) in the body of evidence for each outcome assessed. | Page8-10 |
| **DISCUSSION** | | |  |
| Discussion | 23a | Provide a general interpretation of the results in the context of other evidence. | Page11-13 |
|  | 23b | Discuss any limitations of the evidence included in the review. | Page11-13 |
|  | 23c | Discuss any limitations of the review processes used. | Page11-13 |
|  | 23d | Discuss implications of the results for practice, policy, and future research. | Page11-13 |
| **OTHER INFORMATION** | | |  |
| Registration and protocol | 24a | Provide registration information for the review, including register name and registration number, or state that the review was not registered. | N/A |
|  | 24b | Indicate where the review protocol can be accessed, or state that a protocol was not prepared. | N/A |
|  | 24c | Describe and explain any amendments to information provided at registration or in the protocol. | N/A |
| Support | 25 | Describe sources of financial or non-financial support for the review, and the role of the funders or sponsors in the review. | Authorship form |
| Competing interests | 26 | Declare any competing interests of review authors. | Authorship form |
| Availability of data, code and other materials | 27 | Report which of the following are publicly available and where they can be found: template data collection forms; data extracted from included studies; data used for all analyses; analytic code; any other materials used in the review. | N/A |

**Supplementary Table 3.** PICOS framework

| Population | Patients treated with local salvage therapies for RCC in previously treated kidney, renal fossa, regional lymph nodes, adrenal gland, or psoas muscles |
| --- | --- |
| Intervention | Local therapy to the isolated local recurrence (e.g., surgery, radiotherapy, ablation) |
| Control | Different methods of local recurrence treatment |
| Outcome | Primary: recurrence-free survival  Secondary: overall survival, cancer-specific survival, and rates of major complications |
| Study design | Prospective and retrospective studies |

**Supplementary Table 4.** ROBINS-I

| Study | Classification of intervention | Selection of participants | Deviations from intended interventions | Missing data | Measurement of the outcome | Selection of reported result | Overall |
| --- | --- | --- | --- | --- | --- | --- | --- |
| Candia et al. 2024 [1] | Low | Moderate | Moderate | Low | Moderate | Moderate | Moderate |
| Huang et al. 2023 [2] | Low | Moderate | Moderate | Low | Moderate | Moderate | Moderate |
| Stanik et al. 2022 [3] | Low | Moderate | Low | Moderate | Moderate | Low | Moderate |
| Liu et al. 2022 [4] | Low | Serious | Serious | Low | Moderate | Moderate | Serious |
| Romeo et al. 2020 [5] | Moderate | Moderate | Moderate | Moderate | Moderate | Low | Moderate |
| Herout et al. 2018 [6] | Low | Moderate | Low | Low | Moderate | Low | Moderate |
| Thomas et al. 2015 [7] | Low | Serious | Serious | Low | Low | Low | Serious |
| Fayek et al. 2014 [8] | Low | Serious | Serious | Moderate | Serious | Moderate | Serious |
| Margulis et al. 2009 [9] | Low | Serious | Serious | Low | Low | Low | Serious |
| Bruno et al. 2006 [10] | Low | Moderate | Low | Moderate | Moderate | Low | Moderate |
| Sandhu et al. 2005 [11] | Low | Moderate | Moderate | Moderate | Moderate | Low | Moderate |
| Göğüş et al. 2003 [12] | Low | Moderate | Moderate | Moderate | Moderate | Moderate | Moderate |
| Zhou et al. 2026[13] | Low | Moderate | Moderate | Moderate | Moderate | Moderate | Moderate |
| Porreca et al. 2025 [14] | Serious | Moderate | Serious | Low | Serious | Serious | Serious |
| Ma et al. 2024 [15] | Low | Moderate | Low | Moderate | Moderate | Low | Moderate |
| Okhawere et al. 2023 [16] | Low | Moderate | Low | Moderate | Moderate | Moderate | Moderate |
| Di Maida et al. 2023 [17] | Low | Moderate | Low | Low | Moderate | Moderate | Moderate |
| Huang et al. 2023 [18] | Low | Moderate | Low | Low | Moderate | Low | Moderate |
| Brassetti et al. 2023 [19] | Moderate | Serious | Low | Serious | Moderate | Serious | Serious |
| Brassier et al. 2022 [20] | Low | Low | Low | Moderate | Moderate | Low | Moderate |
| Antonelli et al. 2016 [21] | Moderate | Low | Moderate | Serious | Moderate | Serious | Serious |
| Johnson et al. 2008 [22] | Low | Serious | Moderate | Low | Moderate | Moderate | Serious |
| Hemal et al. 2024 [23] | Low | Moderate | Low | Moderate | Moderate | Moderate | Moderate |
| Margue et al. 2022 [24] | Low | Moderate | Low | Moderate | Moderate | Moderate | Moderate |
| Jimenez et al 2016 [25] | Low | Moderate | Low | Serious | Moderate | Moderate | Serious |
| Gaas et al. 2025 [26] | Low | Moderate | Low | Low | Moderate | Low | Moderate |
| Saini et al. 2023 [27] | Low | Moderate | Low | Moderate | Moderate | Moderate | Moderate |
| Beksac et al. 2021 [28] | Low | Moderate | Low | Serious | Moderate | Serious | Serious |
| Kim et al. 2021 [29] | Low | Moderate | Moderate | Low | Moderate | Moderate | Moderate |
| Barboza et al. 2020 [30] | Low | Moderate | Moderate | Moderate | Moderate | Moderate | Moderate |
| Ghandour et al. 2020 [31] | Low | Moderate | Moderate | Low | Moderate | Low | Moderate |
| Capogrosso et al. 2017 [32] | Low | Moderate | Low | Low | Moderate | Low | Moderate |
| Du et al. 2016 [33] | Low | Moderate | Moderate | Low | Moderate | Low | Moderate |
| Abarzua-Cabezas et al. 2015 [34] | Low | Moderate | Moderate | Moderate | Moderate | Moderate | Moderate |
| Riedl et al. 2011 [35] | Moderate | Serious | Moderate | Serious | Serious | Serious | Serious |
| Vaccaro et al 2025 [36] | Moderate | Moderate | Moderate | Moderate | Moderate | Moderate | Moderate |
| Abdelsalam et al. 2020 [37] | Low | Moderate | Low | Moderate | Moderate | Serious | Serious |
| Zhou et al. 2018 [38] | Low | Moderate | Low | Low | Moderate | Low | Moderate |
| Yang et al. 2013 [39] | Low | Serious | Low | Moderate | Moderate | Moderate | Serious |
| Das et al. 2023 [40] | Moderate | Serious | Moderate | Moderate | Moderate | Moderate | Serious |
| Loloi et al. 2020 [41] | Low | Moderate | Low | Moderate | Moderate | Low | Moderate |
| Sundelin et al. 2019 [42] | Moderate | Moderate | Low | Moderate | Moderate | Moderate | Moderate |
| Yanagisawa et al. 2019 [43] | Low | Moderate | Low | Low | Moderate | Low | Moderate |
| Vijgh R. et al. 2017 [44] | Low | Moderate | Moderate | Moderate | Moderate | Serious | Serious |
| Okhunov et al. 2016 [45] | Low | Moderate | Low | Low | Moderate | Low | Moderate |
| Liu et al. 2025 [46] | Low | Moderate | Low | Low | Moderate | Low | Moderate |
| Krajewski et al. 2024 [47] | Moderate | Serious | Low | Moderate | Serious | Moderate | Serious |

**Supplementary Figure 1. PRISMA 2020 flow diagram for new systematic reviews which included searches of databases, registries and other sources**

Supplementary Figure 2. Sensitivity Analysis for Three-Year Recurrence-Free Survival in Patients Treated With Salvage Surgery for Local Recurrence Following Primary Radical Nephrectomy

Supplementary Figure 3. Five-Year Overall Survival in Patients Treated With Salvage Surgery for Local Recurrence Following Primary Radical Nephrectomy

Supplementary Figure 4. Five-Year Cancer-Specific Survival in Patients Treated With Salvage Surgery for Local Recurrence Following Primary Radical Nephrectomy

Supplementary Figure 5. Meta-regression of 5-year overall survival by publication year in patients treated with salvage surgery for local recurrence following radical nephrectomy

Supplementary Figure 6. Sensitivity Analysis for Rate of Severe or Worse Complications in Patients Treated With Salvage Surgery for Local Recurrence Following Primary Partial Nephrectomy

**Supplementary Appendix 1.** AMSTAR-2 checklist

**Supplementary Appendix 2.** Search strategy

Electronic searches were performed in the following databases to identify eligible studies

1. MEDLINE (via PubMed) (n = 2269)
2. Embase (n = 4400)
3. Web of Science (n = 2274)
4. CENTRAL via Ovid ( n = 410)
5. Google Scholar ( Top 200 hits were retrieved )

No date or language filters were used.

MEDLINE (viaPubMed)

#1 Search: "kidney neoplasms"[MeSH Terms] OR (("renal"[Title/Abstract] OR "kidney*"[Title/Abstract]) AND ("cancer*"[Title/Abstract] OR "carcinoma*"[Title/Abstract] OR "neoplasm*"[Title/Abstract] OR "tumor*"[Title/Abstract] OR "tumour*"[Title/Abstract])) OR "nephrectomy"[Title/Abstract] OR "nephron sparing surgery"[Title/Abstract]
#2 Search: (("Locally recurrent"[Title/Abstract] OR "Locally relapsed"[Title/Abstract] OR "Local recurrence"[Title/Abstract] OR "Local retroperitoneal recurrence"[Title/Abstract] OR "Local relapse"[Title/Abstract] OR "recurrent"[Title/Abstract]) AND ("local"[Title/Abstract] OR "localized"[Title/Abstract] OR "localised"[Title/Abstract])) OR "salvage"[Title/Abstract]
#3 Search: "wilms"[Title/Abstract] OR "nephroblastoma"[Title/Abstract] OR "angiomyolipoma"[Title/Abstract] OR "angiosarcoma"[Title/Abstract] OR "liposarcoma"[Title/Abstract] OR " leiomyosarcoma"[Title/Abstract] OR "adrenal tumor*"[Title/Abstract] OR "urothelial carcinoma"[Title/Abstract] OR "case report"[Title/Abstract]

#4 Search: #1 AND #2 NOT #3

Embase

#1 Search: 'kidney tumor'/exp OR (('renal':ti,ab,kw OR 'kidney*':ti,ab,kw) AND ('cancer*':ti,ab,kw OR 'carcinoma*':ti,ab,kw OR 'neoplasm*':ti,ab,kw OR 'tumor*':ti,ab,kw OR 'tumour*':ti,ab,kw)) OR 'nephrectomy':ti,ab,kw OR 'nephron sparing surgery':ti,ab,kw

#2 Search: (('locally recurrent':ti,ab,kw OR 'locally relapsed':ti,ab,kw OR 'local recurrence':ti,ab,kw OR 'local retroperitoneal recurrence':ti,ab,kw OR 'local relapse':ti,ab,kw OR 'recurrent':ti,ab,kw) AND ('local':ti,ab,kw OR 'localized':ti,ab,kw OR 'localised':ti,ab,kw)) OR 'salvage':ti,ab,kw

#3 Search: 'wilms':ti,ab,kw OR 'nephroblastoma':ti,ab,kw OR 'angiomyolipoma':ti,ab,kw OR 'angiosarcoma':ti,ab,kw OR 'liposarcoma':ti,ab,kw OR 'leiomyosarcoma':ti,ab,kw OR 'adrenal tumor*':ti,ab,kw OR 'urothelial carcinoma':ti,ab,kw OR 'case report':ti,ab,kw

#4 Search: #1 AND #2 NOT #3

Web of Science

#1 TS=((("renal" OR "kidney*") AND ("cancer*" OR "carcinoma*" OR "neoplasm*" OR "tumor*" OR "tumour*")) OR "nephrectomy" OR "nephron sparing surgery")
#2 TS=((("locally recurrent" OR "locally relapsed" OR "local recurrence" OR "local retroperitoneal recurrence" OR "local relapse" OR "recurrent") AND ("local" OR "localized" OR "localised")) OR "salvage")

#3 TS=("wilms" OR "nephroblastoma" OR "angiomyolipoma" OR "angiosarcoma" OR "liposarcoma" OR "leiomyosarcoma" OR "adrenal tumor*" OR "urothelial carcinoma" OR "case report")

#4 #1 AND #2 NOT #3

CENTRAL (via Ovid)

#1 ((("renal" OR "kidney*") AND ("cancer*" OR "carcinoma*" OR "neoplasm*" OR "tumor*" OR "tumour*")) OR "nephrectomy" OR "nephron sparing surgery")
#2 ((("locally recurrent" OR "locally relapsed" OR "local recurrence" OR "local retroperitoneal recurrence" OR "local relapse" OR "recurrent") AND ("local" OR "localized" OR "localised")) OR "salvage")

#3 ("wilms" OR "nephroblastoma" OR "angiomyolipoma" OR "angiosarcoma" OR "liposarcoma" OR "leiomyosarcoma" OR "adrenal tumor*" OR "urothelial carcinoma" OR "case report")

#4 #1 AND #2 NOT #3

Search engine: Google Scholar

(renal | kidney*) (cancer* | carcinoma* | neoplasm* | tumor* | tumour* | nephrectomy | "nephron sparing surgery") ("locally recurrent" | "local recurrence" | salvage)

**References:**

[1]	Candia GAF, Camean J, Jaunarena J, Chemi J, Bachur RV, Villoldo G. Complete laparoscopic management of locally recurrent renal cell carcinoma after radical nephrectomy. Rev Mex Urol 2024;84:1–9. https://doi.org/10.48193/4s2h3424.

[2]	Huang J, Wang Y, Zhang C, Hu X, Wang P, Shi G, et al. Surgical Management and Oncologic Outcomes for Local Retroperitoneal Recurrence of Renal Cell Carcinoma After Radical Nephrectomy. Clin Genitourin Cancer 2023;21:315.e1-315.e7. https://doi.org/10.1016/j.clgc.2022.10.010.

[3]	Staník M, Poprach A, Littnerová S, Čapák I, Hulová M, Šebová N, et al. Oncological outcomes of surgery for isolated retroperitoneal recurrence in renal cancer patients after radical nephrectomy. Urol Oncol 2022;40:111.e27-111.e34. https://doi.org/10.1016/j.urolonc.2021.11.028.

[4]	Liu Y, Zhang X, Ma H, Tian L, Mai L, Long W, et al. Locoregional recurrence after nephrectomy for localized renal cell carcinoma: Feasibility and outcomes of different treatment modalities. Cancer Med 2022;11:4430–9. https://doi.org/10.1002/cam4.4790.

[5]	Romeo A, Garcia Marchiñena P, Jurado AM, Gueglio G. Renal fossa recurrence after radical nephrectomy: Current management, and oncological outcomes. Urol Oncol 2020;38:42.e7-42.e12. https://doi.org/10.1016/j.urolonc.2019.10.004.

[6]	Herout R, Graff J, Borkowetz A, Zastrow S, Leike S, Koch R, et al. Surgical resection of locally recurrent renal cell carcinoma after nephrectomy: Oncological outcome and predictors of survival. Urol Oncol 2018;36:11.e1-11.e6. https://doi.org/10.1016/j.urolonc.2017.08.021.

[7]	Thomas AZ, Adibi M, Borregales LD, Hoang LN, Tamboli P, Jonasch E, et al. Surgical Management of Local Retroperitoneal Recurrence of Renal Cell Carcinoma after Radical Nephrectomy. J Urol 2015;194:316–22. https://doi.org/10.1016/j.juro.2015.02.2943.

[8]	Fayek IS, Habashy HF, Habashy NF. Isolated loco-regional recurrence after radical nephrectomy for renal cell carcinoma: a study of 22 patients. J Egypt Natl Cancer Inst 2014;26:161–6. https://doi.org/10.1016/j.jnci.2014.05.003.

[9]	Margulis V, McDonald M, Tamboli P, Swanson DA, Wood CG. Predictors of oncological outcome after resection of locally recurrent renal cell carcinoma. J Urol 2009;181:2044–51. https://doi.org/10.1016/j.juro.2009.01.043.

[10]	Bruno JJ, Snyder ME, Motzer RJ, Russo P. Renal cell carcinoma local recurrences: impact of surgical treatment and concomitant metastasis on survival. BJU Int 2006;97:933–8. https://doi.org/10.1111/j.1464-410X.2006.06076.x.

[11]	Sandhu SS, Symes A, A’Hern R, Sohaib SAA, Eisen T, Gore M, et al. Surgical excision of isolated renal-bed recurrence after radical nephrectomy for renal cell carcinoma. BJU Int 2005;95:522–5. https://doi.org/10.1111/j.1464-410X.2005.05331.x.

[12]	Göğüş C, Baltaci S, Bedük Y, Sahinli S, Küpeli S, Göğüş O. Isolated local recurrence of renal cell carcinoma after radical nephrectomy: experience with 10 cases. Urology 2003;61:926–9. https://doi.org/10.1016/s0090-4295(02)02582-7.

[13]	Zhou W, Guan H, Yao J, Yang C, Ni W, Liu Y, et al. Pathologic Upstaging in Local Recurrence After Partial Nephrectomy: Distinct Biologic Behavior and Clinical Prognosis of Original-Site Versus Non–Original-Site Recurrence. Ann Surg Oncol 2026. https://doi.org/10.1245/s10434-026-19960-z.

[14]	Porreca A, Marino F, De Marchi D, Giampaoli M, D’Agostino D, Simonetti F, et al. Local Recurrence After Nephron Surgery: What to Do? An Italian Multicentric Registry. Cancers 2025;17:3269. https://doi.org/10.3390/cancers17193269.

[15]	Ma W, Xu W, Meng J, Chen L, Tai S, Yang C, et al. Reoperation for ipsilateral local recurrence following prior nephron-sparing surgery: innovative surgical insights from a high-volume urological center with cross-sectional study. Int J Surg Lond Engl 2025;111:171–7. https://doi.org/10.1097/JS9.0000000000001917.

[16]	Okhawere KE, Grauer R, Zuluaga L, Meilika KN, Ucpinar B, Beksac AT, et al. Operative and oncological outcomes of salvage robotic radical and partial nephrectomy: a multicenter experience. J Robot Surg 2023;17:1579–85. https://doi.org/10.1007/s11701-023-01538-6.

[17]	Di Maida F, Grosso AA, Campi R, Lambertini L, Gallo ML, Cadenar A, et al. Redo Partial Nephrectomy for Local Recurrence After Previous Nephron-sparing Surgery. Surgical Insights and Oncologic Results from a High-volume Robotic Center. Eur Urol Open Sci 2023;57:84–90. https://doi.org/10.1016/j.euros.2023.09.007.

[18]	Huang J, Su R, Zhang C, Bao Y, Hu X, Ye X, et al. Comparative analysis of salvage partial nephrectomy versus radical nephrectomy after the failure of initial partial nephrectomy. Urol Oncol 2023;41:434.e17-434.e25. https://doi.org/10.1016/j.urolonc.2023.07.010.

[19]	Brassetti A, Anceschi U, Bove AM, Cartolano S, Costantini M, Ferriero MC, et al. Redo robotic partial nephrectomy for recurrent renal tumors: Single high-volume center experience. Eur Urol 2023;83:S500–1. https://doi.org/10.1016/S0302-2838(23)00392-5.

[20]	Brassier M, Khene Z-E, Bernhard J-C, Dang VT, Ouzaid I, Nouhaud FX, et al. Percutaneous Ablation Versus Surgical Resection for Local Recurrence Following Partial Nephrectomy for Renal Cell Cancer: A Propensity Score Analysis (REPART Study-UroCCR 71). Eur Urol Focus 2022;8:210–6. https://doi.org/10.1016/j.euf.2021.02.007.

[21]	Antonelli A, Sodano M, Furlan M, Tardanico R, Fisogni S, Carobbio F, et al. MP41-18 PATHOGENESIS, FEATURES AND PROGNOSIS OF RENAL RELAPSE AFTER PARTIAL NEPHRECTOMY. J Urol 2016. https://doi.org/10.1016/j.juro.2016.02.185.

[22]	Johnson A, Sudarshan S, Liu J, Linehan WM, Pinto PA, Bratslavsky G. Feasibility and outcomes of repeat partial nephrectomy. J Urol 2008;180:89–93; discussion 93. https://doi.org/10.1016/j.juro.2008.03.030.

[23]	Hemal A, Refugia J. Robot Assisted Salvage Partial Nephrectomy: A Feasible Option for Management of Tumor Recurrences After Cryoablation or Radiofrequency Ablation. J Endourol 2024;38:A437. https://doi.org/10.1089/end.2024.38524.abstracts.

[24]	Margue G, Michiels C, Allenet C, Dupitout L, Ricard S, Jambon E, et al. Feasibility of salvage robotic partial nephrectomy after ablative treatment failure (UroCCR-62 study). Minerva Urol Nephrol 2022;74:209–15. https://doi.org/10.23736/S2724-6051.22.04693-6.

[25]	Jiménez JA, Zhang Z, Zhao J, Abouassaly R, Fergany A, Gong M, et al. Surgical Salvage of Thermal Ablation Failures for Renal Cell Carcinoma. J Urol 2016;195:594–600. https://doi.org/10.1016/j.juro.2015.09.078.

[26]	Gaas MYa, Kaprin AD, Kostin AA, Epifanova MV, Vorobyev NV, Inozemtsev RO, et al. Surgical treatment of local renal cell carcinoma recurrence. Surgical aspects and oncological results. Sib J Oncol 2025;24:15–28. https://doi.org/10.21294/1814-4861-2025-24-1-15-28.

[27]	Saini S, O’Rourke T, Thakker P, Hemal A. V05-09 ROBOT-ASSISTED PARTIAL NEPHRECTOMY FOR NEW OR RECURRENT TUMOR IN PATIENT WITH PRIOR PARTIAL NEPHRECTOMY OR TUMOR ABLATION. J Urol 2023. https://doi.org/10.1097/JU.0000000000003263.09.

[28]	Beksac AT, Carbonara U, Abou Zeinab M, Meagher M, Hemal S, Tafuri A, et al. Redo Robotic Partial Nephrectomy for Recurrent Renal Tumors: A Multi-Institutional Analysis. J Endourol 2022;36:1296–301. https://doi.org/10.1089/end.2021.0954.

[29]	Kim SH, Choi MG, Shin JH, Kim Y-A, Chung J. A Real-World, Population-Based Retrospective Analysis of Therapeutic Survival for Recurrent Localized Renal Cell Carcinoma After Nephrectomy. Front Oncol 2021;11:693831. https://doi.org/10.3389/fonc.2021.693831.

[30]	Barboza MP, Speir RW, Boris RS, Calaway AC, Cary C, Foster RS, et al. Understanding the role of salvage lymphadenectomy in node only recurrences after nephrectomy for renal cell carcinoma. Urol Oncol 2020;38:739.e1-739.e8. https://doi.org/10.1016/j.urolonc.2020.06.006.

[31]	Ghandour R, Miranda AF, Singla N, Meng X, Enikeev D, Woldu S, et al. Feasibility and Safety of Robotic Excision of Ipsilateral Retroperitoneal Recurrence After Nephrectomy for Renal Cell Carcinoma. Urology 2020;145:159–65. https://doi.org/10.1016/j.urology.2020.07.060.

[32]	Capogrosso P, Barret E, Nunes-Silva I, Sanchez-Salas R, Rozet F, Larcher A, et al. MP16-15 NATURAL HISTORY OF SURGICALLY TREATED LOCAL RECURRENCE AFTER NEPHRECTOMY. J Urol 2017. https://doi.org/10.1016/j.juro.2017.02.521.

[33]	Du Y, Grüllich C, Hadaschik B, Hatiboglu G, Hohenfellner M, Pahernik S. Local Recurrence After Curative Surgical Treatment of Renal Cell Cancer: A Study of 91 Patients. Clin Genitourin Cancer 2016;14:e379-385. https://doi.org/10.1016/j.clgc.2016.01.012.

[34]	Abarzua-Cabezas FG, Sverrisson E, De La Cruz R, Spiess PE, Haddock P, Sexton WJ. Oncological and functional outcomes of salvage renal surgery following failed primary intervention for renal cell carcinoma. Int Braz J Urol Off J Braz Soc Urol 2015;41:147–54. https://doi.org/10.1590/S1677-5538.IBJU.2015.01.20.

[35]	Riedl, Pahernik, Habl, Hohenfellner. UP-01.204 Surgery + Intraoperative Radiotherapy (IORT) + Additive External Beam Radiation: A Promising Option for Treatment of Locally Recurrent Renal Cell Carcinoma (RCC)? Urology 2011;2088:A1. https://doi.org/10.1016/j.urology.2011.07.754.

[36]	Vaccaro C, Mistretta FA, Piccinelli ML, Lievore E, Jannello LMI, Fontana M, et al. Thermal ablation for local tumor recurrence after previous partial nephrectomy: Perioperative and oncological outcomes. Urol Oncol 2025;43:444.e1-444.e10. https://doi.org/10.1016/j.urolonc.2025.03.018.

[37]	Abdelsalam M, Sabir S, Hudspeth T, Kusin S, Matin S, Wood C, et al. 3:27 PM Abstract No. 267 Efficacy of percutaneous image-guided thermal ablation for renal cell carcinoma following ipsilateral partial nephrectomy. J Vasc Interv Radiol 2020;31:S119–20. https://doi.org/10.1016/j.jvir.2019.12.315.

[38]	Zhou W, Herwald SE, Uppot RN, Arellano RS. Image-Guided Thermal Ablation for Non-resectable Recurrence of Renal Cell Cancer Following Nephrectomy: Clinical Experience with Eleven Patients. Cardiovasc Intervent Radiol 2018;41:1743–50. https://doi.org/10.1007/s00270-018-1976-2.

[39]	Yang B, Autorino R, Remer EM, Laydner HK, Hillyer S, Altunrende F, et al. Probe ablation as salvage therapy for renal tumors in von Hippel-Lindau patients: the Cleveland Clinic experience with 3 years follow-up. Urol Oncol 2013;31:686–92. https://doi.org/10.1016/j.urolonc.2011.05.008.

[40]	Das A, Shapiro D, Borza T, Wells S, Hinshaw L, Ziemlewicz T, et al. PD40-06 COMPARING ONCOLOGIC OUTCOMES BETWEEN SURGERY AND MICROWAVE ABLATION FOR RENAL MASSES BETWEEN 3 AND 7 CM. J Urol 2023. https://doi.org/10.1097/JU.0000000000003345.06.

[41]	Loloi J, Shingleton WB, Nakada SY, Zagoria RJ, Landman J, Lee BR, et al. Management of Residual or Recurrent Disease Following Thermal Ablation of Renal Cortical Tumors. J Kidney Cancer VHL 2020;7:1–5. https://doi.org/10.15586/jkcvhl.2020.133.

[42]	Sundelin MO, Lagerveld B, Ismail M, Keeley FX, Nielsen TK. Repeated Cryoablation as Treatment Modality After Failure of Primary Renal Cryoablation: A European Registry for Renal Cryoablation Multinational Analysis. J Endourol 2019;33:909–13. https://doi.org/10.1089/end.2019.0444.

[43]	Yanagisawa T, Miki J, Sano T, Otsuka T, Enei Y, Iwatani K, et al. PD51-11 UTILITY OF SALVAGE PERCUTANEOUS CRYOABLATION FOR LOCALLY RECURRENCE RCC AFTER PRIMARY CRYOABLATION. J Urol 2019.

[44]	Van Der Vijgh R, Montauban Van Swijndregt A, Lagerveld B, Sandkuyl R. The course of recurrence following cryoablation for renal tumors. J Endourol 2017;31:A281. https://doi.org/10.1089/end.2017.29029.abstracts.

[45]	Okhunov Z, Chamberlin J, Moreira DM, George A, Babaian K, Shah P, et al. Salvage Percutaneous Cryoablation for Locally Recurrent Renal-Cell Carcinoma After Primary Cryoablation. J Endourol 2016;30:632–7. https://doi.org/10.1089/end.2016.0088.

[46]	Liu R, Liu Y, Zhang Z, Gao J, Zhang X, Pan Q, et al. 2648P Phase II clinical study of the safety and efficacy of Sintilimab in combination with Axitinib and stereotactic radiotherapy in the treatment of local-regional recurrent renal cell carcinoma. Ann Oncol 2025;36:S1327. https://doi.org/10.1016/j.annonc.2025.08.3261.

[47]	Krajewski W, Guziński M, Tomczak W, Nowak Ł, Łaszkiewicz J, Chorbińska J, et al. Percutaneous cryoablation of kidney tumors after partial nephrectomy. Videosurgery Miniinvasive Tech n.d.;19:483–8. https://doi.org/10.20452/wiitm.2024.17904.
